# Comparative genomics and transcriptomics on salt tolerance of *Vigna luteola*

**DOI:** 10.1270/jsbbs.25042

**Published:** 2026-02-04

**Authors:** Yurie Iki, Fanmiao Wang, Kosuke Ito, Takanori Wakatake, Keitaro Tanoi, Ken Naito

**Affiliations:** 1 Graduate School of Frontier Sciences, The University of Tokyo, 5-1-5 Kashiwanoha, Kashiwa, Chiba 277-8561, Japan; 2 Research Center of Genetic Resources, National Agriculture and Food Research Organization, 2-1-2 Kannondai, Tsukuba, Ibaraki 305-8602, Japan; 4 Graduate School of Agricultural and Life Sciences, The University of Tokyo, 1-1-1 Yayoi, Bunkyo, Tokyo 113-8657, Japan; 5 Fukushima Institute for Research, Education and Innovation (F-REI), 6-1 Yazawa-machi, Gongendo, Namie-town, Fukushima 979-1521, Japan

**Keywords:** hairy cowpea, salt tolerance, transcriptome, *Vigna luteola*, wild species

## Abstract

*Vigna luteola*, a wild legume species, shows remarkable variation in salinity tolerance across its natural habitats, with coastal populations exhibiting high tolerance and riverbank populations being sensitive. This intraspecific variation provides a valuable system for investigating the genetic basis of salt tolerance. A major QTL for salt tolerance was previously identified by crossing salt-tolerant and salt-sensitive accessions, but the responsible genes remain unknown. In this study, grafting experiments between the two accessions revealed that the root plays a primary role in salt tolerance by suppressing Na^+^ transport to the shoot. We then conducted root transcriptome analysis and identified four candidate genes located within the QTL and highly expressed under salt stress in the tolerant accession: *CBL-INTERACTING PROTEIN KINASE 6 (CIPK6)*, *CAFFEOYL SHIKIMATE ESTERASE (CSE)*, *FCS-LIKE ZINC FINGER PROTEIN 13 (FLZ13)*, and *DROUGHT-INDUCED 21 (DI21)*. Promoter analysis revealed that the *CIPK6* promoter contains transcription factor binding motifs unique to the salt-tolerant accession, which may contribute to its high expression under salt stress. These findings suggest that *CIPK6* is regulated by *cis*-regulatory differences and is the most promising candidate for the salt-tolerance QTL. The identified genes in this study provide a foundation for developing salt-tolerant crops in the future.

## Introduction

Groundwater depletion is a serious problem in world agriculture. As many of the food-exporting countries rely on groundwater for irrigation, it significantly impacts nations that rely on imported products for their staple food supply (Dalin *et al.* 2017, Wada *et al.* 2010). Thus, it is urgent to develop salt-tolerant crops to realize agriculture with saline water.

Although hundreds of genes potentially involved in salt tolerance have been identified, it remains unclear how to utilize them effectively. Model plants, which are generally salt-sensitive, are useful for identifying salt tolerance–related genes, for example, by isolating salt-sensitive mutants and determining the underlying genetic changes. However, such studies do not reveal which combinations of these genes would be the most effective. For example, screening the best combination of two genes out of those related to salt tolerance, the possible combinations could reach several hundred thousand. In addition, they do not clarify in which specific organs or tissues these genes should be expressed or the optimal timing for their expression. This gap is critical because there is often a trade-off between salt tolerance and growth (Liang *et al.* 2024), and constitutive overexpression of salt tolerance–related genes can cause undesirable effects.

Instead, we may find a direct answer in wild species that are adapted to a saline environment. As one such species, we have focused on *Vigna luteola* (Jacq.) Benth. It mainly lives on tropical marine beaches in West Africa and the Americas, and some accessions can survive a condition of 400 mM NaCl for more than 4 weeks (Yoshida *et al.* 2020). Interestingly, some populations of this species have settled in riverbanks and are not salt tolerant (Noda *et al.* 2025, Yoshida *et al.* 2020). A previous study crossed the salt-tolerant and salt-sensitive accessions of *V. luteola* and identified a single major QTL involved in salt tolerance (Chankaew *et al.* 2014). Other previous studies revealed that the salt-tolerant accession of *V. luteola* allocates more Na^+^ in the root (Noda *et al.* 2022, Wang *et al.* 2024), whereas the salt-sensitive one allocates more Na^+^ in the shoot (Noda *et al.* 2025, Wang *et al.* 2024). In addition, the salt-tolerant accession of *V. luteola* consistently allocates Na^+^ to the topmost fully expanded leaves, suggesting the “taking turns” system of Na^+^-loading leaves (Noda *et al.* 2022). These results indicate that the salt-tolerant accession possesses distinct salt-tolerant mechanisms in the root and shoot.

However, it remains unclear whether the root contributes more to salt tolerance or the shoot does. Moreover, the genes responsible for salt tolerance in *V. luteola* have not yet been identified. Thus, in this study, we first conducted grafting experiments between the salt-tolerant and salt-sensitive accessions and examined their salt tolerance and Na^+^/K^+^ allocation. Our results suggest that the root plays a more critical role in salt tolerance, particularly by suppressing Na^+^ transport to the shoot. Based on these findings, we analyzed the root transcriptome data (Noda *et al.* 2025) and identified four candidate genes that met the following three criteria: (1) located within the major QTL region related to salt tolerance, (2) highly expressed in the salt-tolerant accession under salt stress, and (3) homologous to genes with functions potentially contributing to salt tolerance. Promoter regions of the four candidate genes were then analyzed to identify differences in *cis*-regulatory motifs potentially involved in the different expression patterns between the two accessions. As a result, *CIPK6* was considered the most promising candidate gene for root-mediated salt tolerance mechanisms. The candidate genes identified in this study could be targets for developing salt-tolerant crops in the future.

## Materials and Methods

### Plant materials

Two accessions of *Vigna luteola* were used: a salt-tolerant accession, JP233389, which inhabits beaches, and a salt-sensitive accession, JP235855, which inhabits riverbanks. These seeds were obtained from the Research Center of Genetic Resources, National Agriculture and Food Research Organization (NARO), Japan (NARO Genebank: https://www.gene.affrc.go.jp/index_en.php). It should be noted that in the previous studies of our laboratory prior to 2018, including the QTL mapping study by (Chankaew *et al.* 2014), the salt-tolerant accession of *Vigna luteola* (JP233389) was referred to as *Vigna marina* subsp. *oblonga* (Chankaew *et al.* 2014, Iseki *et al.* 2016, 2018, Takahashi *et al.* 2016). But according to the study by Pasquet (2001), which classified *V. marina* subsp. *oblonga* as *V. luteola*, NARO Genebank corrected the species name of JP233389 as *V. luteola*. Moreover, Chankaew *et al.* (2014) mislabeled the tolerant accession as JP235855 and the sensitive accession as JP233389. However, the genetic identities of the accessions used in our study were the same as those used in Chankaew *et al.* (2014).

Hereafter, the salt-tolerant accession of *V. luteola* (JP233389) is referred to as the tolerant accession, and the salt-sensitive accession of *V. luteola* (JP235855) is referred to as the sensitive accession.

Plants were grown as described previously (Wang *et al.* 2024). Briefly, germinated seeds were transplanted into a hydroponic solution and grown in a growth chamber. The hydroponic solution was used at double the concentration compared to Wang *et al.* (2024) to minimize nutritional deficiency stress during the salt tolerance test and during ion concentration analysis. Specifically, we used OAT House No.1 and OAT House No.2 (OAT Agrio Co., Ltd., Tokyo, Japan) at double the standard concentrations, resulting in final concentrations of 37.2 mEq L^–1^ N, 10.2 mEq L^–1^ P, 17.2 mEq L^–1^ K, 16.4 mEq L^–1^ Ca, and 6.0 mEq L^–1^ Mg.

### Methods of grafting experiments

Germinated seedlings were transplanted to a hydroponic culture as described above. After 1–2 days, seedlings were cut in a V-shape (Supplemental Fig. 1) and grafted using 12.5 mm wide Micropore surgical tape (3M Japan Limited, Tokyo, Japan). Four grafting combinations (scion/rootstock) were produced: tolerant/tolerant (T/T), tolerant/sensitive (T/S), sensitive/tolerant (S/T), and sensitive/sensitive (S/S). Shoots and roots were taken from different plants even if they were the same accessions (T/T or S/S). After grafting, plants were returned to the original hydroponic culture and were initially maintained in a high-humidity environment after grafting to prevent desiccation. The grafting success rate was approximately 80%. Plants that successfully grafted and showed similar growth stages were selected for the following experiments.

### Salt tolerance test of grafted plants

Five plants were grafted with each combination (T/T, S/T, T/S, S/S) using the methods described above. Three T/S plants and four plants from the other grafting combinations were selected at a comparable growth stage, when the third leaf was approximately fully expanded. These plants were then transferred to the hydroponic culture containing 200 mM NaCl. After 5, 8, 12, and 15 days of salt treatment, wilt scores were evaluated by three to four persons using a scale of 1, 3, 5, 7, and 9, corresponding to 0%, 25%, 50%, 75%, and 100% wilting, respectively.

### Analysis of Na^+^ and K^+^ allocation

The methods followed those described previously by Noda *et al.* (2022) with minor modifications. Briefly, germinated plants of the tolerant and sensitive accessions were transferred to the hydroponic culture containing double the standard concentrations of OAT House No.1 and OAT House No.2. After the first leaf fully expanded, six replicates were treated with or without 200 mM NaCl in the hydroponic culture for three days. Subsequently, shoots and roots were sampled, dried, and digested with 60% and 69% HNO3, respectively. Then, the sodium (Na) and potassium (K) contents were measured using inductively coupled plasma-mass spectrometry (ICP-MS, NexION 350S, PerkinElmer, Waltham, MA, USA).

Grafted plants were also subjected to the same experiments. At least 10 plants were generated for each grafting combination (T/T, S/T, T/S, S/S) using the methods described above. Four plants from each grafting combination were selected at the same growth stage, when the third leaf was fully expanded. These plants were then transferred to the hydroponic culture containing 200 mM NaCl. And Na and K contents of shoots and roots were measured as described above. The graft sites, covered with surgical tape, were not sampled due to the difficulty of removing the tape.

### DNA extraction, genome sequence, and assembly of the sensitive accession

Genome sequencing and assembly for the tolerant accession JP233389 were previously conducted by Noda *et al.* (2025). Therefore, in this study, we did genome sequencing and assembly only for the sensitive accession.

As described in (Naito 2023), high molecular weight DNA was extracted from unexpanded leaves with the NucleoBond HMW DNA kit (MACHEREY-NAGEL GmbH & Co. KG, Düren, Germany), size-selected with Short Read Eliminator XL kit (Pacific Biosciences of California, Inc., Menlo Park, CA, USA). Sequencing libraries for nanopore sequencing were prepared with SQK-LSK110 (Oxford Nanopore Technologies KK, Tokyo, Japan). Each library was then loaded to PromethION R9.4.1 Flowcell (Oxford Nanopore Technologies KK) and was run for 72 h. The obtained raw data was transformed into fastq format with guppy-5.0.7. Short reads were also obtained with HiSeq 4000 (Illumina, San Diego, CA, USA), which was provided as a customer service by GeneBay, Inc. (Yokohama, Japan).

Draft assembly was performed with necat-0.0.1 (Chen *et al.* 2021) with default parameters except “PREP_OUTPUT_COVERAGE=60” and “CNS_OUTPUT_COVERAGE=40”. Polishing was done with racon-1.4.3 (Vaser *et al.* 2017) and medaka-1.0.3 (https://github.com/nanoporetech/medaka) twice each. Further polishing was also done with hypo-1.0.3 using short reads. Scaffolds were generated using RagTag (Alonge *et al.* 2022) using the genome of the tolerant accession as a reference. Any controversies between the contigs and linkage maps were regarded as misassemblies and manually fixed as described by Sakai *et al.* (2015). Genome completeness of the assembly was evaluated using BUSCO (5.8.3) (Manni *et al.* 2021) with the fabales_odb12 database.

### Gene model prediction

To obtain mRNA-seq data for gene model prediction, seeds of the tolerant accession (JP233389) were sown in pots filled with culture soil and grown in a greenhouse. Whole shoots and whole roots were sampled from three plants of 3-week-old and total RNA was extracted. The RNA was sequenced with Hiseq 2000 (Illumina K.K., Tokyo, Japan). Library construction and sequencing were provided as a custom service of GeneBay, Inc. These mRNA-seq data differ from the 3ʹmRNA-seq data used for the transcriptome analysis described below.

Gene model predictions for the two accessions (JP233389, JP235855) of *V. luteola* were accomplished by integrating the results of Braker2 and GeMoMa. For Braker2 prediction, repetitive elements in assembled genome sequences were first soft-masked using RepeatMasker (4.1.2) (https://github.com/rmhubley/RepeatMasker) with the accession-specific repeat sequence library generated by RepeatModeler (2.0.3) (https://github.com/Dfam-consortium/RepeatModeler). Next, an ab initio gene model prediction was performed on the soft-masked genome with Braker2 (2.1.5) (Brůna *et al.* 2021) and GeneMark (Brůna *et al.* 2020) using the “odb10_plant” protein database from OrthoDB (Kriventseva *et al.* 2019) for protein homology information. Gene models showing homology with the database were selected using a script (https://github.com/Gaius-Augustus/BRAKER/tree/report/scripts/predictionAnalysis) and utilized for subsequent analyses. For GeMoMa analysis, *de novo* transcriptome assembly was first conducted with Trinity (2.13.2) (Grabherr *et al.* 2011) using quality-filtered full-length RNA-seq reads from root and leaf tissues of the tolerant accession. Default parameters were applied for assembly. Subsequently, the same RNA-seq reads were mapped to the *de novo* transcriptome assembly using HISAT2 (2.2.1) (Kim *et al.* 2019), and mapped reads were retrieved using samtools (1.15.1) (Danecek *et al.* 2021). These reads were utilized for intron and UTR prediction during the GeMoMa process. Homology-based gene prediction was performed by transferring the high-quality gene model of *V. marina* (https://doi.org/10.1101/2022.03.28.486085) using GeMoMa (1.8) (Keilwagen *et al.* 2019). The Braker2 prediction was integrated with the GeMoMa results using gffcompare (0.12.6) (Pertea and Pertea 2020). Gene models with the class code “u” were added to the GeMoMa result. Functional gene annotation was carried out on the resulting gene models using EnTAP (0.10.8) (Hart *et al.* 2020). TrEMBL and SwissProt (https://www.uniprot.org/) and NCBI plant RefSeq (https://ncbi.nlm.nih.gov/refseq/) were employed as databases for homology search. Unannotated gene model entries were removed from the final gene models. Finally, gene models were renamed using GeMoMa annotation finalizer. The quality of the final gene models was assessed using BUSCO (5.8.3) (Manni *et al.* 2021) against fabales_odb12.

### Determining the QTL region in the genome of the tolerant accession

The genome of the tolerant accession JP233389 (Noda *et al.* 2025) was used for this analysis. Chankaew *et al.* (2014) identified the major QTL related to salt tolerance, located at 101.6 cM of chromosome 1 between the marker CEDG087 and cp06039. We performed BLASTN (Camacho *et al.* 2009) using the marker sequence as the query and a custom database, created from the FASTA file of chromosome 1, as the subject.

### Differential expression analysis

Root transcriptome data obtained by 3ʹ mRNA-seq from the tolerant (JP233389) and sensitive (JP235855) accessions (Noda *et al.* 2025) were used for transcriptome analysis. Read counts for each gene were estimated using Salmon (1.10.3) (Patro *et al.* 2017) with default parameters. In this process, the transcriptome assembly of the tolerant accession was used as the reference. The obtained count data was used for differential expression analysis using edgeR (4.0.11) (Robinson *et al.* 2010). We followed the protocol of the case study (Fu *et al.* 2015) in the edgeR user’s guide. We compared the expression levels of each gene in the tolerant accession under salt stress and the sensitive accession under salt stress at the same time point. ANOVA-like tests were not performed; instead, differential expression tests were conducted separately for each group.

Genes meeting the following criteria in at least two of the four time points were selected as differentially expressed genes (DEGs): logFC > 0 (higher in the tolerant accession) with *p*-value < 0.05 and FDR < 0.05, or logFC < 0 (lower in the tolerant accession) with *p*-value < 0.05 and FDR < 0.05.

### Gene clustering with self-organizing maps for candidate gene selection

DEGs were clustered with self-organizing maps (SOM) (Wehrens and Buydens 2007), following the protocol described in this script (https://iamciera.github.io/SOMexample/). Several map sizes, including 4 × 4, 4 × 5, and 6 × 6, were tested to determine the optimal map size. The optimal map size was selected by considering the clarity of the generated clusters.

Clusters showing high expression in the tolerant accession under salt stress and low expression in the sensitive accession under all conditions were used to identify candidate genes.

### Homology search and amino acid comparison

A Homology search was performed with DIAMOND (Buchfink *et al.* 2021) blastp with option “--max-target-seqs 1” and “--evalue 1e-5”. Protein sequences of *A. thaliana* were obtained from The Arabidopsis Information Resource (TAIR) (https://www.arabidopsis.org/download_files/Proteins/Araport11_protein_lists/Araport11_pep_20220914_representative_gene_model.gz). Gene Ontology (GO) annotations of *Arabidopsis thaliana* were downloaded from TAIR (https://www.arabidopsis.org/download_files/GO_and_PO_Annotations/Gene_Ontology_Annotations/ATH_GO_GOSLIM.txt.gz), and genes related to salt stress or abscisic acid (ABA) were identified based on GO terms or gene descriptions. Specifically, GO terms containing “salinity”, “salt”, or “sodium” were classified as related to salt stress (Supplemental Table 1). Those containing “abscisic acid” were classified as related to abscisic acid (Supplemental Table 2).

Amino acid sequences of selected genes were aligned using ClustalW (2.1) (Larkin *et al.* 2007) with the slow/accurate pairwise alignment option. Additionally, some sequences were analyzed with InterPro (Paysan-Lafosse *et al.* 2023) to detect domain positions using the default options on the website.

### Promoter analysis of four candidate genes

We extracted 2.5 kb regions upstream of the transcription start site (TSS) of candidate genes from both tolerant and sensitive accessions. The extracted promoter sequences were analyzed using FIMO in the MEME Suite (Grant *et al.* 2011) with Arabidopsis DAP motifs (O’Malley *et al.* 2016) to identify potential binding motifs. The identified motifs were then compared between the accessions to identify those unique to either the tolerant or sensitive accession.

Subsequently, the promoters of cluster 14 genes and other genes (used as a control) from the tolerant accession were analyzed using FIMO, as described above. The number of genes containing tolerant-specific motifs—identified in candidate gene promoters—was counted for each group. A binomial distribution test was conducted to compare the proportions of motif-containing genes between cluster 14 and the control group. Additionally, the relative frequencies of motifs significantly enriched in cluster 14 were analyzed across the promoter regions and compared with those in the control group.

### Data availability

The RNA sequence data is available at the Sequence Read Archive of the NCBI website. The BioProject ID is PRJNA1080052. BioSamples IDs range from SAMN40099594 to SAMN40099689, but only samples containing “R1”, “R2”, and “R3” in the Sample Name were used in this study.

## Results

### Salt tolerance test of grafted plants

**Fig. 1.**
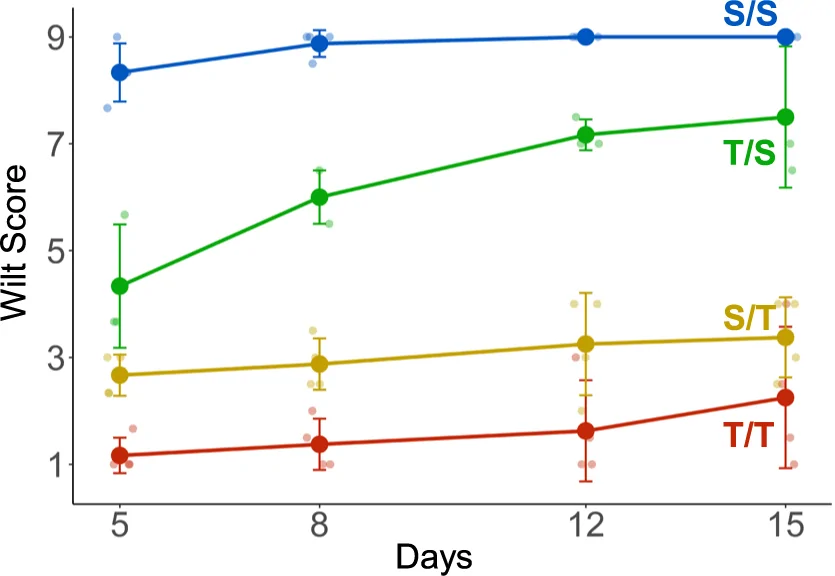
The salt tolerance test of grafted plants under 200 mM NaCl. Small, pale-colored points represent the wilt scores of individual plants. The larger points indicate mean values for each line. Error bars indicate standard deviations. T/T, S/T, S/S, and T/S indicate the scion/rootstock combinations. T and S represent the tolerant and sensitive accessions, respectively.

To elucidate the importance of the root and shoot on salt tolerance, we generated grafted plants using the tolerant (T) and sensitive (S) accessions. The salt tolerance test of grafted plants showed that T/T (scion/rootstock) plants had the lowest wilt score, followed by S/T plants and T/S plants, while S/S plants had the highest wilt score (Fig. 1). S/S plants almost completely wilted only five days after salt treatment. Initially, T/S plants maintained significantly lower wilt scores than S/S plants, however, they got high wilt scores 15 days after salt treatment. In contrast, T/T and S/T plants maintained low wilt scores even 15 days after salt treatment. These results suggest that the root of the tolerant accession plays a critical role in conferring salt tolerance in *V. luteola*.

### Na^+^ and K^+^ allocation in the two accessions

**Fig. 2.**
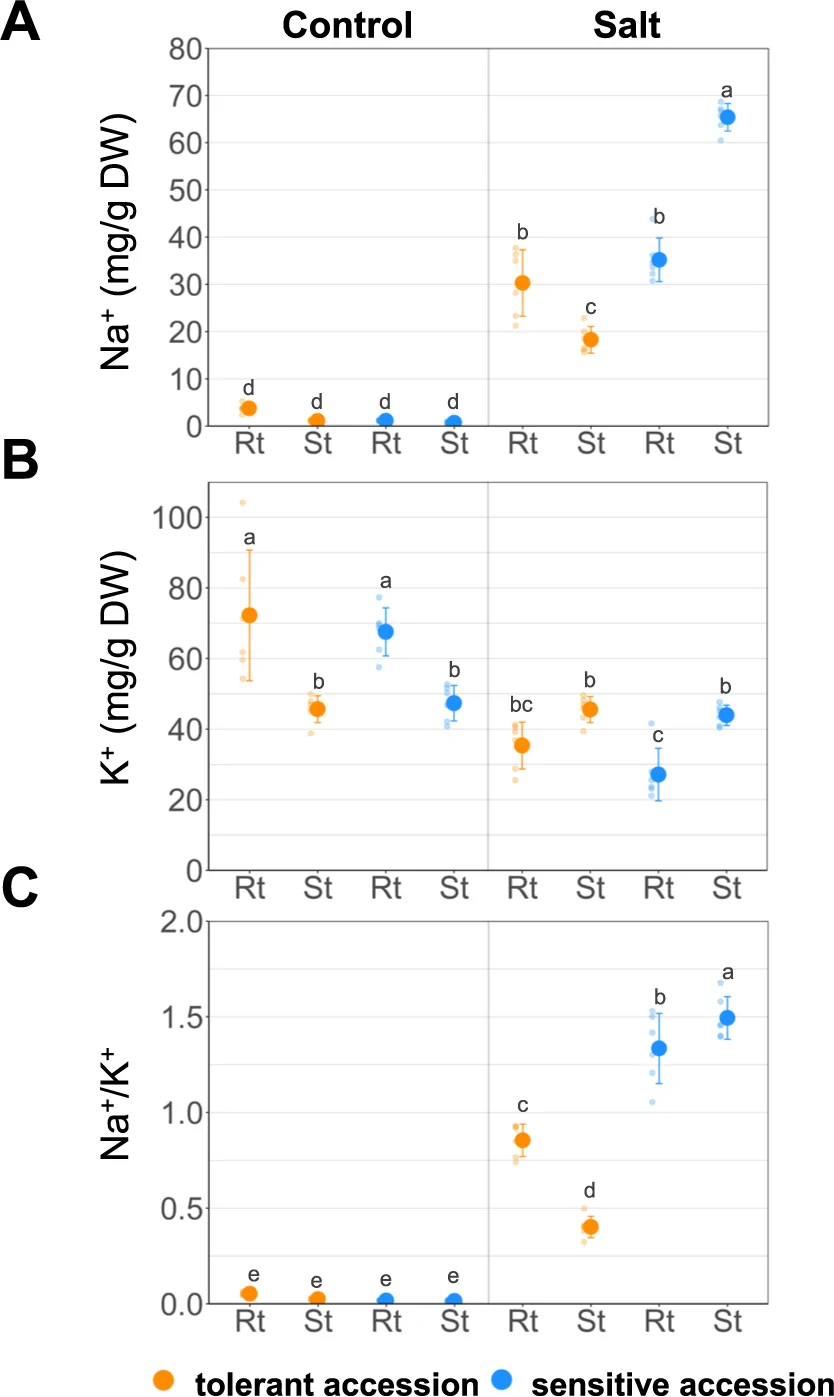
Na^+^ and K^+^ concentrations and the Na^+^/K^+^ ratio in roots (Rt) and shoots (St) of the tolerant and sensitive accessions under control and salt (200 mM NaCl) treatment for 3 days. (A) Na^+^ concentration (mg/g DW). (B) K^+^ concentration (mg/g DW). (C) Na^+^/K^+^ ratio. The small, pale-colored points represent values from individual root and shoot samples (n = 6). Larger points and error bars represent the mean and standard deviation, respectively. Different letters above the plots indicate significant differences determined by the Tukey’s HSD test (*p* < 0.05).

We examined Na^+^ and K^+^ concentrations in whole shoots and roots under both salt and control conditions to determine the role of the root in Na^+^/K^+^ homeostasis. Under control conditions, Na^+^ concentrations were quite low compared to salt conditions, but the root of the tolerant accession accumulated the most Na^+^ (Fig. 2A). K^+^ concentrations in the shoot were not significantly different between the two accessions, with both maintaining approximately 45 mg/gDW (Fig. 2B). Similar to Na^+^ concentration, Na^+^/K^+^ ratios were quite low under control conditions, but the root of the tolerant accession had the highest ratio (Fig. 2C).

Under salt stress, Na^+^ concentrations in the shoot were significantly higher in the sensitive accession compared to the tolerant accession. In contrast, Na^+^ concentration in the root under salt stress did not differ significantly between the two accessions. Notably, the tolerant accession showed higher Na^+^ concentration in the root than the shoot, whereas the sensitive accession exhibited the opposite pattern, with higher Na^+^ concentrations in the shoot than in the root (Fig. 2A). Shoot K^+^ concentrations did not differ significantly between the two accessions, with both maintaining approximately 45 mg/g DW, similar to the control conditions. The root K^+^ concentration was slightly lower in the sensitive accession than in the tolerant one but not significant (Fig. 2B). The Na^+^/K^+^ ratio was considerably lower in the tolerant accession compared to the sensitive accession, particularly in the shoot (Fig. 2C). These results suggest that the tolerant accession maintains a lower Na^+^/K^+^ ratio in the shoot by suppressing Na^+^ accumulation in the shoot.

### Na^+^ and K^+^ allocation in the grafted plants

**Fig. 3.**
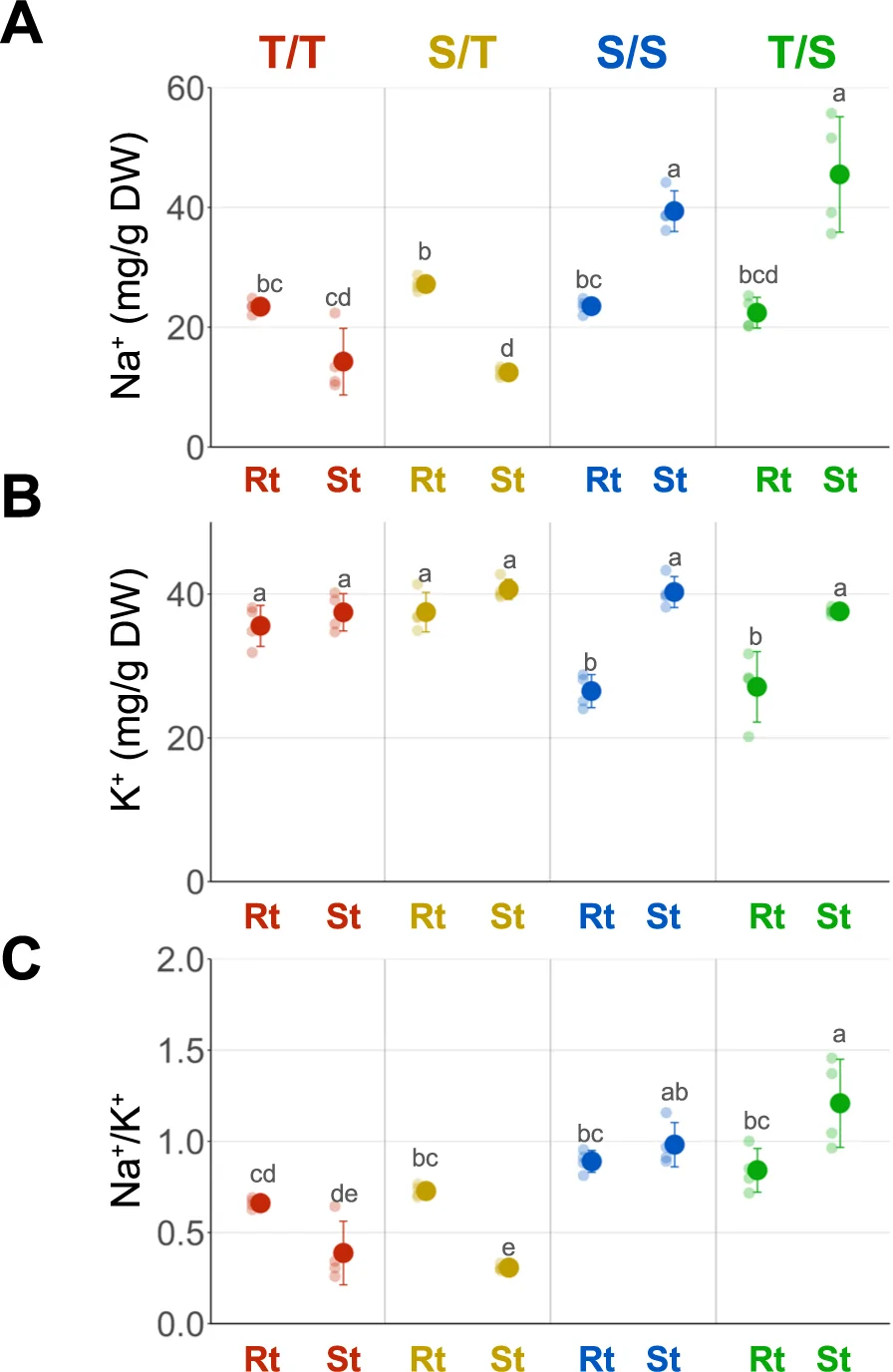
Na^+^ and K^+^ concentrations and the Na^+^/K^+^ ratio in roots (Rt) and shoots (St) of grafted plants under salt (200 mM NaCl) treatment for three days. (A) Na^+^ concentration (mg/g DW). (B) K^+^ concentration (mg/g DW). (C) Na^+^/K^+^ ratio. Small, pale-colored points represent values from individual root and shoot samples (n = 4). Larger points and error bars indicate the mean and standard deviation, respectively. Different letters above the plots indicate significant differences determined by the Tukey’s HSD test (*p* < 0.05). T/T, S/T, S/S, and T/S indicate the scion/rootstock combinations. T and S represent the tolerant and the sensitive accessions, respectively.

To test whether Na^+^ accumulation in the shoot is controlled by the root or the shoot, we analyzed Na^+^ and K^+^ allocation in grafted plants under salt stress. Grafted plants with the same rootstock exhibited similar trends: T/T and S/T showed similar trends, while S/S and T/S also showed similar trends (Fig. 3). For example, shoot Na^+^ concentrations were significantly lower in T/T and S/T than in S/S and T/S, although root Na^+^ concentrations showed no substantial differences among T/T, S/T, S/S, and T/S (Fig. 3A). Both T/T and S/T showed lower Na^+^ concentrations in the shoot than in the root. In contrast, S/S and T/S exhibited higher Na^+^ concentrations in the shoot than in the root.

Shoot K^+^ concentration did not differ among T/T, S/T, S/S, and T/S, although the root K^+^ concentration was lower in S/S and T/S than in T/T and S/T (Fig. 3B). Shoot Na^+^/K^+^ ratio was significantly lower in T/T and S/T compared to S/S and T/S (Fig. 3C). Moreover, T/T and S/T showed a lower Na^+^/K^+^ ratio in the shoot than in the root. In contrast, S/S and T/S showed a higher Na^+^/K^+^ ratio in the shoot than in the root. These results suggest that the root plays a primary role in suppressing Na^+^ transport to the shoot.

### Genome assembly and determining the physical position of the QTL region

For QTL localization and comparison of amino acid and promoter sequences between the two accessions, we re-annotated the genome of the tolerant accession (Noda *et al.* 2025) and newly assembled the genome of the sensitive accession. We sequenced the whole genome of the sensitive accession using Oxford Nanopore. The draft genome assembly contained 496.2 Mbp in 210 contigs with an N50 length of 11.5 Mbp. These contigs were further scaffolded into 11 scaffolds (Supplemental Tables 3, 4). BUSCO analysis (v5.8.3, fabales_odb12) of the assembly showed 98.4% completeness in the sensitive accession. For comparison, we re-analyzed the genome assembly of the tolerant accession (Noda *et al.* 2025) using the same version and database, which showed 98.5% completeness. Gene annotation for the two accessions predicted 33,814 genes in the sensitive accession and 31,191 genes in the tolerant accession, covering 98.5% and 97.8% of BUSCO genes (v5.8.3, fabales_odb12), respectively.

Subsequently, we identified the genomic region corresponding to the major QTL for salt tolerance, previously identified by Chankaew *et al.* (2014), in the tolerant accession genome. The major QTL region ranged from 57,314,479 bp to 62,876,770 bp on chromosome 1 and contained 557 genes (Supplemental Table 5).

### Gene expression analysis and the candidate genes

We performed gene expression analysis under salt stress to further narrow down candidate genes. First, we identified differentially expressed genes (DEGs) between the two accessions under salt stress using the root transcriptome data (Noda *et al.* 2025). The number of genes expressed more in the tolerant accession was 1,709 in the first daytime (1D), 4,021 in the first nighttime (1N), 2,901 in the second daytime (2D), and 3,808 in the second nighttime (2N). The number of genes expressed less in the tolerant accession was 1,652 in 1D, 4,111 in 1N, 2,484 in 2D, and 3,915 in 2N (Supplemental Fig. 2). A total of 6,917 genes detected at least 2 time points were used for further clustering.

**Fig. 4.**
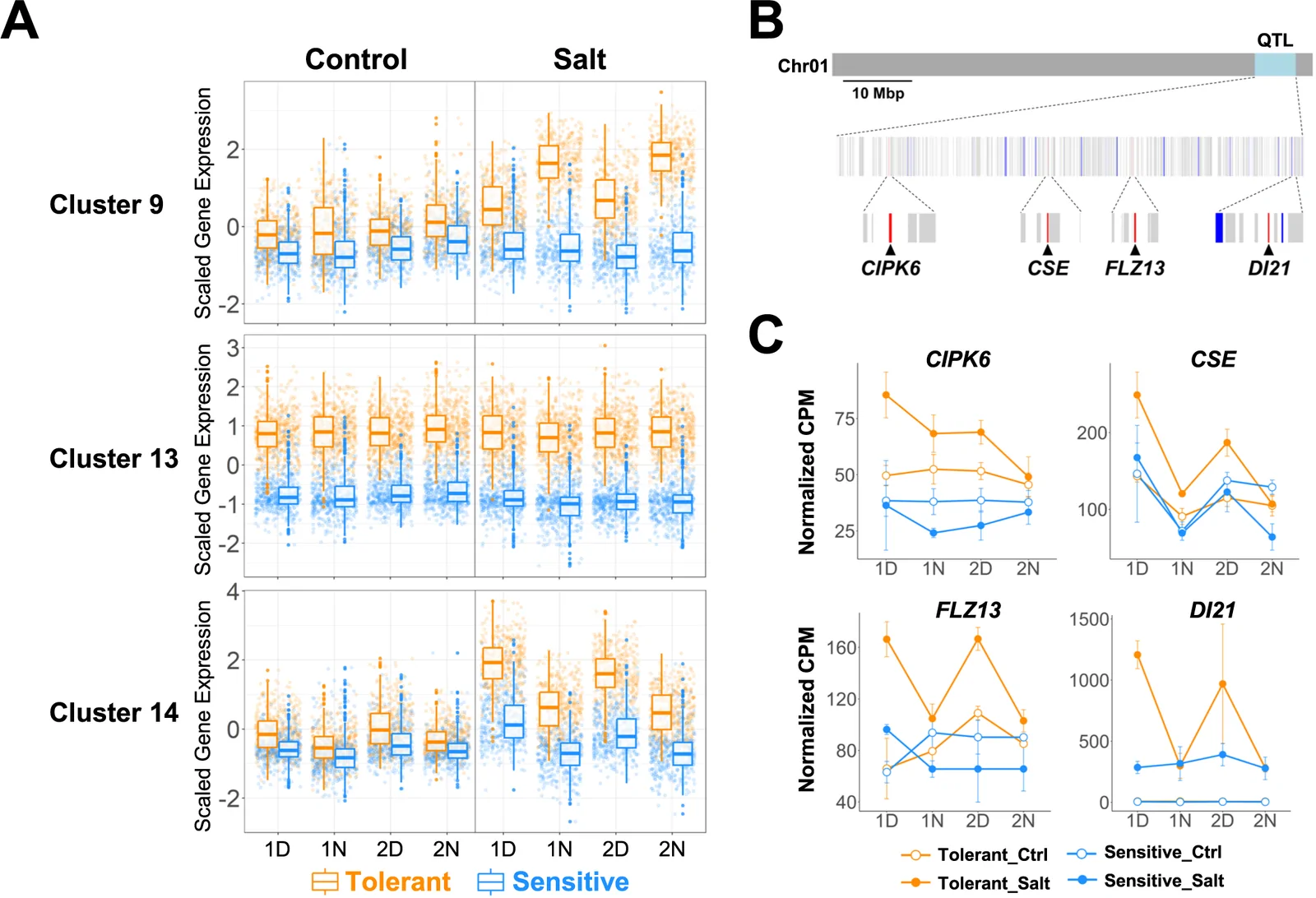
Expression patterns of candidate clusters and the candidate genes. (A) Expression patterns of the three candidate clusters that are expressed more in the tolerant accession under salt stress. Tolerant and Sensitive indicate the tolerant and sensitive accessions, respectively. Control and Salt indicate control (0 mM NaCl) and salt stress (200 mM NaCl) conditions, respectively. 1D, 1N, 2D, and 2N represent 1st daytime, 1st nighttime, 2nd daytime, and 2nd nighttime, respectively. (B) Genomic positions of genes within the QTL region and candidate clusters. The light blue region indicates the major QTL (*Saltol1.1*) region associated with salt tolerance. Vertical bars represent individual genes within the QTL region. Genes belonging to the candidate clusters are shown in blue, while the four candidate genes (*CIPK6*, *CSE*, *FLZ13*, and *DI21*) are shown in red. Other genes in the region are shown in gray. *CIPK6*, *FLZ13*, *CSE*, and *DI21* indicate *Viglu256296.01G0396300.1*, *Viglu256296.01G0413000.1*, *Viglu256296.01G0424800.1*, and *Viglu256296.01G0445600.1*, respectively. (C) Expression levels of the four candidate genes. The dots and error bars indicate the mean and standard deviation of the biological replicates of normalized CPM, respectively. Tolerant and Sensitive indicate the tolerant accession and the sensitive accession, respectively. Ctrl and Salt indicate control and salt stress conditions, respectively. Tolerant_Ctrl is not visible because it overlaps with Sensitive_Ctrl due to identical values in *DI21*.

SOM-clustering divided the DEGs into 16 clusters (Supplemental Fig. 3). Among the 16 clusters, we focused on three clusters—9, 13, and 14—that showed high expression under salt stress in the tolerant accession (Fig. 4A). These clusters contained 375 genes (cluster 9), 526 genes (cluster 13), and 401 genes (cluster 14). In the three candidate clusters, 27 genes were in the QTL region (Supplemental Fig. 4, Supplemental Table 6). Among those, 25 genes had Arabidopsis homologs, whereas the remaining two genes contained no detectable domain (Supplemental Table 6). Four genes were selected for further analysis by GO terms (Supplemental Tables 1, 2) or gene function related to salt tolerance (Fig. 4B).

The four genes were *Viglu256296.01G0396300.1*, homologous to *AT4G30960.1 CBL-INTERACTING PROTEIN KINASE 6* (*CIPK6*), with the GO term “response to salt stress”; *Viglu256296.01G0413000.1*, homologous to *AT1G52760.1 CAFFEOYL SHIKIMATE ESTERASE* (*CSE*), reported to be involved in lignin biosynthesis; *Viglu256296.01G0424800.1*, homologous to *AT1G74940.1 FCS-LIKE ZINC FINGER PROTEIN 13* (*FLZ13*), with the GO term “response to salt stress”; and *Viglu256296.01G0445600.1*, homologous to *AT4G15910.1 DROUGHT-INDUCED 21* (*DI21*), with the GO term “response to abscisic acid”. All four genes belonged to cluster 14 (Fig. 4A) and were highly expressed in the tolerant accession under salt stress, particularly during the daytime (Fig. 4C).

A comparison of the amino acid sequences of the tolerant accession and the sensitive accession showed that CIPK6 and FLZ13 had insertion-deletion mutations and substitutions (Supplemental Figs. 5, 6), whereas DI21 and CSE were entirely identical (Supplemental Fig. 7). However, the mutations were not within the conserved domains (Supplemental Figs. 5, 6). These findings suggest that the protein functions of the four candidate genes are highly conserved between the two accessions, and that their differential expression may contribute to salt tolerance in the tolerant accession.

### Promoter analysis on *CIPK6*, *CSE*, *FLZ13*, and *DI21*

We next examined the promoter regions to explore potential *cis*-regulatory differences. *CIPK6*, *CSE*, *FLZ13*, and *DI21* are located within the major QTL region, but it remains unclear whether their expression is regulated by *cis*-regulatory elements in the QTL. To investigate this, we analyzed promoter sequences for differences in transcription factor binding sites (TFBSs) between the two accessions. Because the relative distance from the TSS is known to influence gene expression in plants (Voichek *et al.* 2024), we also examined the positional distribution of TFBSs.

**Fig. 5.**
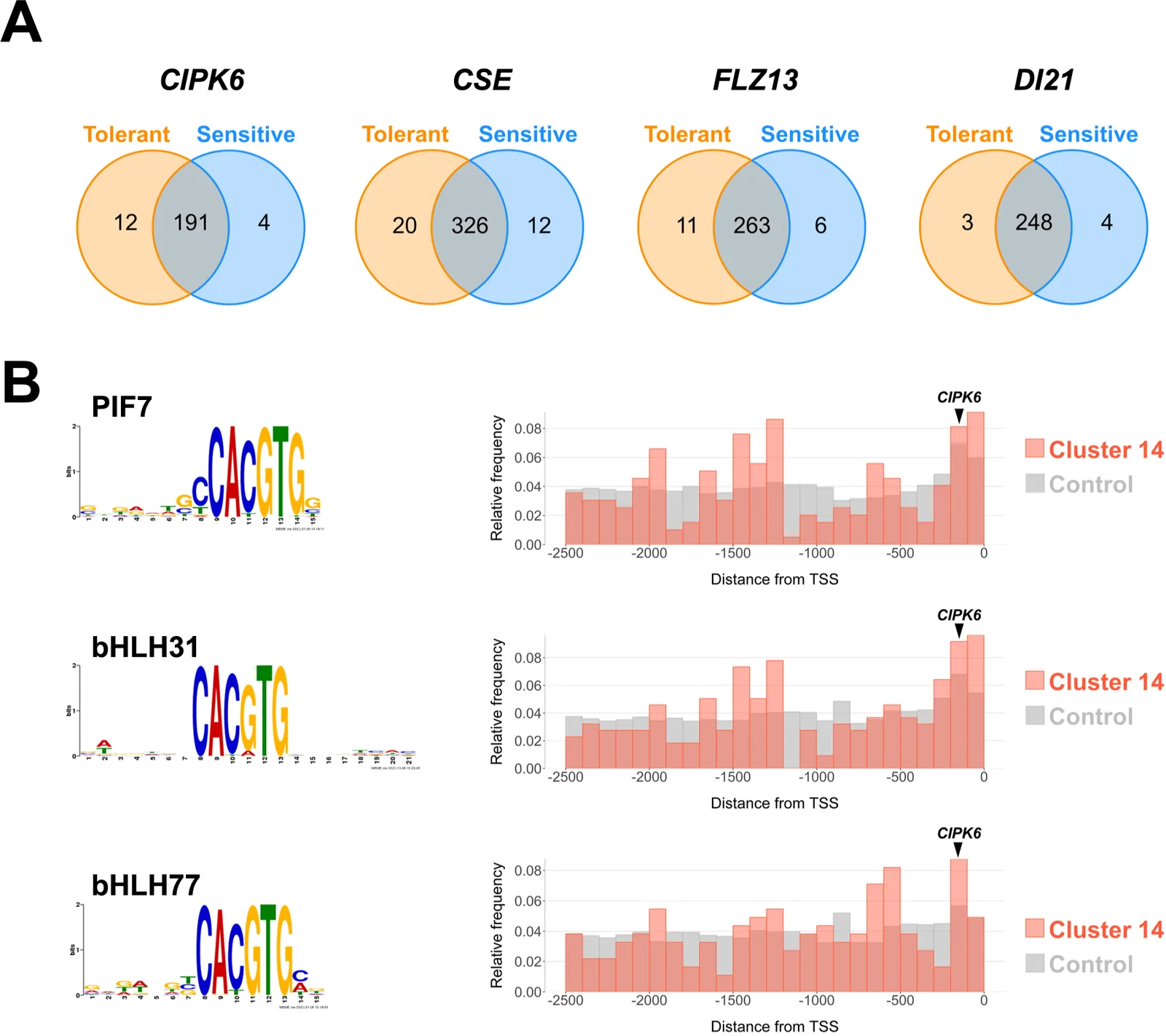
Promoter analysis of the candidate genes. (A) Number of unique and shared motifs of the promoters (2.5 kb upstream from TSS) in the two accessions. (B) Transcription factor binding motifs unique to the tolerant accession in the *CIPK6* promoter, enriched in cluster 14 promoters compared to control, and are located at positions corresponding to the motif-enriched regions in cluster 14 promoters. Pictograms of the motifs and relative frequencies of these motifs across the promoters of cluster 14 and control groups are shown. Black arrows indicate the positions of the motifs on the *CIPK6* promoter.

First, promoter regions of the four genes were analyzed through motif scanning to identify potential binding motifs. As a result, 12, 20, 11, and 3 distinct motifs unique to the tolerant accession were identified in the promoters of *CIPK6*, *CSE*, *FLZ13*, and *DI21*, respectively (Fig. 5A, Supplemental Fig. 8).

To investigate whether these motifs are associated with gene expression patterns, we also analyzed the promoters of cluster 14 genes and other genes (used as control). We identified motifs that were significantly more frequent in the promoter regions of genes in cluster 14 compared to those in the control gene set, based on a binomial distribution test. As a result, the promoters of *CIPK6*, *CSE*, *FLZ13*, and *DI21* contained 3, 11, 3, and 1 such motifs, respectively (Supplemental Table 7). In addition, investigation of the position from TSS revealed that binding motifs for PIF7, bHLH31, and bHLH77 were enriched within 200 bp upstream of the TSS in the promoters of cluster 14, corresponding to the location of the *CIPK6* promoter (Fig. 5B). However, no such motifs were detected in the promoters of *CSE*, *FLZ13*, and *DI21* at positions corresponding to the motif-enriched regions in cluster 14 promoters (Supplemental Fig. 9).

Furthermore, we investigated whether the transcription factors *PIF7*, *bHLH31*, and *bHLH77* also belonged to cluster 14. As a result, we found that *Viglu256296.01G0358900.3*, a homolog of *bHLH31* (*BPEp*, *AT1G59640.2*), was present in cluster 14 and showed similar expression patterns with *CIPK6* (Supplemental Fig. 10A). Amino acid comparison showed that the bHLH_AtBPE_like domain region is highly conserved between Viglu256296.01G0358900.3 and AT1G59640.2 (Supplemental Fig. 10B).

## Discussion

In this study, we demonstrated that suppressing Na^+^ transport to the shoot plays a major role in the salt tolerance of the tolerant accession of *V. luteola*. By integrating root transcriptome data and QTL, we narrowed down the candidate genes for the luteola’s salt tolerance, including functionally important genes such as *CIPK6*, *CSE*, *FLZ13*, and *DI21*. The promoter analysis revealed that a *cis*-element within 200 bp upstream of *CIPK6* is associated with induced expression under salt stress.

### Suppressing Na^+^ transport to the shoot is a primary factor in salt tolerance

Given that S/T plants showed lower wilt scores than T/S plants, the root should play a more important role in the salt tolerance of *V. luteola* (Fig. 1). This, together with the fact that grafted plants with rootstock of the tolerant accession significantly suppressed Na^+^ allocation to the shoot (Fig. 3A), indicates that the root of the tolerant accession has higher ability to block Na^+^ transport. In contrast, the grafted plants did not show significant differences in K^+^ allocation to the shoot (Fig. 3B). Thus, the suppression of Na^+^ transport in the tolerant accession does not rely on increased K^+^ uptake. This indicates that the mechanism of *V. luteola* is different from many other salt-tolerant plants that, in response to salt stress, increase the K^+^ content to prevent Na^+^ uptake (Ito *et al.* 2024, Kumari *et al.* 2021, Noda *et al.* 2022). Previously, we have elucidated that the tolerant accession of *V. luteola* suppresses Na^+^ transport by lignification of Casparian strip (Wang *et al.* 2025) and excludes Na^+^ from the root by increasing the expression of *Salt Overly Sensitive 1* (*SOS1*) that encodes a Na^+^/H^+^ antiporter (Noda *et al.* 2025). As the *SOS1* locus is not located in the QTL region, the genes within the QTL could be involved in upregulating the *SOS1* transcription.

In addition, although the T/S and S/S plants allocated a similar amount of Na^+^ to the shoot, the S/S plants showed a higher wilt score (Figs. 1, 3). This indicates that the shoot of the tolerant accession can tolerate higher concentrations of tissue Na^+^. Although the contribution is smaller than the mechanism in the root, the difference is still significant and will be worth investigating.

### *CIPK6* is the most promising candidate gene for the QTL

The results of this study indicate that *CIPK6* is the most promising candidate gene for salt tolerance. It is located within the previously identified QTL (Chankaew *et al.* 2014), strongly induced by salt stress (Fig. 4C), and has a *cis*-element variation (Fig. 5B) potentially involved in its upregulation by salt stress. *CIPK6* encodes CBL-interacting protein kinase (CIPK), which is activated through its interaction with a calcium sensor, calcineurin B-like protein (CBL) (Batistič and Kudla 2009, He *et al.* 2013). The CBL–CIPK network plays important roles under various stresses (Yu *et al.* 2014). Furthermore, in a previous study of rice, overexpression of *CdtCIPK5*, an ortholog of *AtCIPK6*, enhanced salt tolerance by restricting Na^+^ transport to the shoot (Huang *et al.* 2020). Similarly, the tolerant accession showed low Na^+^ accumulation in the shoot (Fig. 2). Therefore, we consider that *CIPK6* may contribute to suppressing Na^+^ transport in the tolerant accession of *V. luteola*. The conserved amino acid sequences in the functional domains suggest that the CIPK6 protein has similar functions in both accessions (Supplemental Fig. 5), and we hypothesize that the observed difference in expression explains the difference in salt tolerance. The *CIPK6* promoter in the tolerant accession harbors binding sites for bHLH transcription factors including PIF7, bHLH31, and bHLH77. These motifs were significantly enriched in the promoters of cluster 14 genes, which were highly expressed under salt stress in the tolerant accession (Fig. 4A). Moreover, these motifs were located within 200 bp upstream from the TSS in *CIPK6*, the same position where they are frequently enriched in cluster 14 promoters (Fig. 5B). Since the distance of transcription factor binding sites (TFBSs) from the TSS is known to influence gene expression (Voichek *et al.* 2024), this positional overlap suggests that these motifs may contribute to the salt-induced expression of *CIPK6*. Notably, a homolog of *bHLH31* showed a similar expression pattern to *CIPK6*, and the bHLH_AtBPE_like domain was highly conserved between *V. luteola* and *Arabidopsis* (Supplemental Fig. 10). These observations support the possibility that increased expression of *bHLH31* in the tolerant accession may drive the higher expression of *CIPK6* under salt stress, though it remains to be tested whether these motifs directly regulate *CIPK6* expression. Overall, these results suggest that polymorphisms in the *CIPK6* promoter might be a causal variant for the major QTL.

### Other candidate genes

Besides *CIPK6*, we identified *CSE*, *FLZ13*, and *DI21* as possible candidates. Although no notable differences in *cis*-regulatory motifs associated with gene expression were observed (Supplemental Fig. 9), these genes are also located within the QTL, highly expressed in the tolerant accession, and possess functions potentially related to salt tolerance.

*DI21* would contribute to water uptake under salt stress rather than Na^+^ transport. In previous studies, *DI21* has been up-regulated by progressive drought stress (Gosti *et al.* 1995, Yan *et al.* 2012). Salt stress is known to be associated with osmotic stress (Zhang *et al.* 2006). The tolerant accession of *V. luteola* exhibited higher water uptake under salt stress compared to the sensitive accession, although not statistically significant (Wang *et al.* 2024). While the precise function of *DI21* remains unclear, this gene would contribute to water uptake rather than suppress Na^+^ transport under salt stress.

We consider that *CSE* would contribute to lignification in the Casparian strip formation, thereby preventing Na^+^ transport to the shoot in the root. Previous research has reported that *CSE* is involved in lignin biosynthesis (Vanholme *et al.* 2013). Moreover, disruption of lignification in the root increased Na^+^ transport to the shoot in the tolerant accession of *V. luteola* (Wang *et al.* 2025). Based on these reports, *CSE* is considered to play a role in suppressing Na^+^ transport to the shoot in the root through lignin biosynthesis.

*FLZ13* has been reported to interact with *Abscisic acid insensitive5* (*ABI5*) and positively regulate the ABA signaling (Yang *et al.* 2023). ABA signaling is known to play a significant role in drought and salt stress responses (Zhang *et al.* 2006). Although the precise role of *FLZ13* in salt tolerance remains unclear, it is also considered to contribute to the salt stress response.

Interestingly, the expression of the four candidate genes—*CIPK6*, *CSE*, *FLZ13*, and *DI21*—was up-regulated during the daytime while down-regulated during the nighttime under salt stress in the tolerant accession (Fig. 4C). During the daytime, salt stress is more severe because of sodium uptake in a transpiration-dependent manner (Dinneny 2015), in addition to higher temperature and light intensity. Indeed, the tolerant accession of *V. luteola* has been reported to exclude more sodium during the daytime, likely due to daytime-specific up-regulation of *Salt Overly Sensitive 2* (*SOS2*), a kinase involved in *SOS1* activation (Noda *et al.* 2025). Therefore, it is reasonable that genes related to salt stress or drought stress were up-regulated during the daytime to mitigate severe stress.

In addition to the four candidate genes discussed above, the QTL region harbors 23 genes that also exhibited higher expression in the tolerant accession under salt stress (Supplemental Table 6). As we cannot currently exclude the possibility that some of them are also involved in salt tolerance, we need further investigation to identify the responsible gene.

### Conclusions and future perspectives

In conclusion, our findings indicate that suppression of Na^+^ transport to the shoot by the root is essential for salt tolerance in *V. luteola*. Additionally, the high expression of *CIPK6* in the root of the tolerant accession is considered to play a key role in this mechanism. To validate this hypothesis, future complementation studies will be necessary. The candidate genes identified in this study have the potential to contribute to the development of salt-tolerant crops.

## Author Contribution Statement

YI and KN conceived the study. YI produced grafting plants and performed a salt tolerance test. YI, FW, and KT performed ion measurements. KI, FW, and KN performed sequencing and genome assembly. TW performed gene model prediction. YI, FW, and KN analyzed transcriptome data. YI and KN performed promoter analysis. YI and KN wrote the paper.

## Supplementary Material

Supplemental Figures

Supplemental Tables

## References

[B1] Alonge, M., L. Lebeigle, M. Kirsche, K. Jenike, S. Ou, S. Aganezov, X. Wang, Z.B. Lippman, M.C. Schatz and S. Soyk (2022) Automated assembly scaffolding using RagTag elevates a new tomato system for high-throughput genome editing. Genome Biol 23: 258. 36522651 10.1186/s13059-022-02823-7 PMC9753292

[B2] Batistič, O. and J. Kudla (2009) Plant calcineurin B-like proteins and their interacting protein kinases. Biochim Biophy Acta Mol Cell Res 1793: 985–992. 10.1016/j.bbamcr.2008.10.006 19022300

[B3] Brůna, T., A. Lomsadze and M. Borodovsky (2020) GeneMark-EP+: eukaryotic gene prediction with self-training in the space of genes and proteins. NAR Genom Bioinform 2: lqaa026. 32440658 10.1093/nargab/lqaa026 PMC7222226

[B4] Brůna, T., K.J. Hoff, A. Lomsadze, M. Stanke and M. Borodovsky (2021) BRAKER2: automatic eukaryotic genome annotation with GeneMark-EP+ and AUGUSTUS supported by a protein database. NAR Genom Bioinform 3: lqaa108. 33575650 10.1093/nargab/lqaa108 PMC7787252

[B5] Buchfink, B., K. Reuter and H.-G. Drost (2021) Sensitive protein alignments at tree-of-life scale using DIAMOND. Nat Methods 18: 366–368. 33828273 10.1038/s41592-021-01101-x PMC8026399

[B6] Camacho, C., G. Coulouris, V. Avagyan, N. Ma, J. Papadopoulos, K. Bealer and T.L. Madden (2009) BLAST+: architecture and applications. BMC Bioinformatics 10: 421. 20003500 10.1186/1471-2105-10-421 PMC2803857

[B7] Chankaew, S., T. Isemura, K. Naito, E. Ogiso-Tanaka, N. Tomooka, P. Somta, A. Kaga, D.A. Vaughan and P. Srinives (2014) QTL mapping for salt tolerance and domestication-related traits in *Vigna marina* subsp. *oblonga*, a halophytic species. Theor Appl Genet 127: 691–702. 24370961 10.1007/s00122-013-2251-1

[B8] Chen, Y., F. Nie, S.-Q. Xie, Y.-F. Zheng, Q. Dai, T. Bray, Y.-X. Wang, J.-F. Xing, Z.-J. Huang, D.-P. Wang et al. (2021) Efficient assembly of nanopore reads via highly accurate and intact error correction. Nat Commun 12: 60. 33397900 10.1038/s41467-020-20236-7 PMC7782737

[B9] Dalin, C., Y. Wada, T. Kastner and M.J. Puma (2017) Groundwater depletion embedded in international food trade. Nature 543: 700–704. 28358074 10.1038/nature21403 PMC7427584

[B10] Danecek, P., J.K. Bonfield, J. Liddle, J. Marshall, V. Ohan, M.O. Pollard, A. Whitwham, T. Keane, S.A. McCarthy, R.M. Davies et al. (2021) Twelve years of SAMtools and BCFtools. Gigascience 10: giab008. 33590861 10.1093/gigascience/giab008 PMC7931819

[B11] Dinneny, J.R. (2015) Traversing organizational scales in plant salt-stress responses. Curr Opin Plant Biol 23: 70–75. 25449729 10.1016/j.pbi.2014.10.009

[B12] Fu, N.Y., A.C. Rios, B. Pal, R. Soetanto, A.T.L. Lun, K. Liu, T. Beck, S.A. Best, F. Vaillant, P. Bouillet et al. (2015) EGF-mediated induction of Mcl-1 at the switch to lactation is essential for alveolar cell survival. Nat Cell Biol 17: 365–375. 25730472 10.1038/ncb3117

[B13] Gosti, F., N. Bertauche, N. Vartanian and J. Giraudat (1995) Abscisic acid-dependent and -independent regulation of gene expression by progressive drought in *Arabidopsis thaliana*. Mol Gen Genet 246: 10–18. 7823904 10.1007/BF00290128

[B14] Grabherr, M.G., B.J. Haas, M. Yassour, J.Z. Levin, D.A. Thompson, I. Amit, X. Adiconis, L. Fan, R. Raychowdhury, Q. Zeng et al. (2011) Full-length transcriptome assembly from RNA-Seq data without a reference genome. Nat Biotechnol 29: 644–652. 21572440 10.1038/nbt.1883 PMC3571712

[B15] Grant, C.E., T.L. Bailey and W.S. Noble (2011) FIMO: scanning for occurrences of a given motif. Bioinformatics 27: 1017–1018. 21330290 10.1093/bioinformatics/btr064 PMC3065696

[B16] Hart, A.J., S. Ginzburg, M.S. Xu, C.R. Fisher, N. Rahmatpour, J.B. Mitton, R. Paul and J.L. Wegrzyn (2020) EnTAP: Bringing faster and smarter functional annotation to non-model eukaryotic transcriptomes. Mol Ecol Resour 20: 591–604. 31628884 10.1111/1755-0998.13106

[B17] He, L., X. Yang, L. Wang, L. Zhu, T. Zhou, J. Deng and X. Zhang (2013) Molecular cloning and functional characterization of a novel cotton CBL-interacting protein kinase gene (*GhCIPK6*) reveals its involvement in multiple abiotic stress tolerance in transgenic plants. Biochem Biophys Res Commun 435: 209–215. 23660187 10.1016/j.bbrc.2013.04.080

[B18] Huang, S., M. Chen, Y. Zhao, X. Wen, Z. Guo and S. Lu (2020) CBL4-CIPK5 pathway confers salt but not drought and chilling tolerance by regulating ion homeostasis. Environ Exp Bot 179: 104230.

[B19] Iseki, K., Y. Takahashi, C. Muto, K. Naito and N. Tomooka (2016) Diversity and evolution of salt tolerance in the genus *Vigna*. PLoS One 11: e0164711. 27736995 10.1371/journal.pone.0164711 PMC5063378

[B20] Iseki, K., Y. Takahashi, C. Muto, K. Naito and N. Tomooka (2018) Diversity of drought tolerance in the genus *Vigna*. Front Plant Sci 9: 729. 29963062 10.3389/fpls.2018.00729 PMC6014140

[B21] Ito, M., H. Ohashi, M. Takemoto, C. Muto, T. Seiko, Y. Noda, E. Ogiso-Tanaka, A.J. Nagano, Y. Takahashi, J. Furukawa et al. (2024) Single candidate gene for salt tolerance of *Vigna nakashimae* (Ohwi) Ohwi & Ohashi identified by QTL mapping, whole genome sequencing and triplicated RNA-seq analyses. Breed Sci 74: 93–102. 39355622 10.1270/jsbbs.23053 PMC11442111

[B22] Keilwagen, J., F. Hartung and J. Grau (2019) GeMoMa: homology-based gene prediction utilizing intron position conservation and RNA-seq data. Methods Mol Biol 2019: 161–177. 10.1007/978-1-4939-9173-0_9 31020559

[B23] Kim, D., J.M. Paggi, C. Park, C. Bennett and S.L. Salzberg (2019) Graph-based genome alignment and genotyping with HISAT2 and HISAT-genotype. Nat Biotechnol 37: 907–915. 31375807 10.1038/s41587-019-0201-4 PMC7605509

[B24] Kriventseva, E.V., D. Kuznetsov, F. Tegenfeldt, M. Manni, R. Dias, F.A. Simão and E.M. Zdobnov (2019) OrthoDB v10: sampling the diversity of animal, plant, fungal, protist, bacterial and viral genomes for evolutionary and functional annotations of orthologs. Nucleic Acids Res 47: D807–D811. 30395283 10.1093/nar/gky1053 PMC6323947

[B25] Kumari, S., H. Chhillar, P. Chopra, R.R. Khanna and M.I.R. Khan (2021) Potassium: A track to develop salinity tolerant plants. Plant Physiol Biochem 167: 1011–1023. 34598021 10.1016/j.plaphy.2021.09.031

[B26] Larkin, M.A., G. Blackshields, N.P. Brown, R. Chenna, P.A. McGettigan, H. McWilliam, F. Valentin, I.M. Wallace, A. Wilm, R. Lopez et al. (2007) Clustal W and Clustal X version 2.0. Bioinformatics 23: 2947–2948. 17846036 10.1093/bioinformatics/btm404

[B27] Liang, X., J. Li, Y. Yang, C. Jiang and Y. Guo (2024) Designing salt stress-resilient crops: Current progress and future challenges. J Integr Plant Biol 66: 303–329. 38108117 10.1111/jipb.13599

[B28] Manni, M., M.R. Berkeley, M. Seppey, F.A. Simão and E.M. Zdobnov (2021) BUSCO update: Novel and streamlined workflows along with broader and deeper phylogenetic coverage for scoring of eukaryotic, prokaryotic, and viral genomes. Mol Biol Evol 38: 4647–4654. 34320186 10.1093/molbev/msab199 PMC8476166

[B29] Naito, K. (2023) How to sequence and assemble plant genomes. Methods Mol Biol 2632: 57–77. 36781721 10.1007/978-1-0716-2996-3_5

[B30] Noda, Y., R. Sugita, A. Hirose, N. Kawachi, K. Tanoi, J. Furukawa and K. Naito (2022) Diversity of Na^+^ allocation in salt-tolerant species of the genus *Vigna*. Breed Sci 72: 326–331. 36699821 10.1270/jsbbs.22012 PMC9868329

[B31] Noda, Y., F. Wang, S. Chankaew, H. Ariga, C. Muto, Y. Iki, H. Ohashi, Y. Takahashi, H. Sakai, K. Iseki et al. (2025) Diurnal regulation of SOS pathway and sodium excretion underlying salinity tolerance of *Vigna marina*. Plant Cell Environ 48: 3925–3938. 39853734 10.1111/pce.15402 PMC12050389

[B32] O’Malley, R.C., S.-S.C. Huang, L. Song, M.G. Lewsey, A. Bartlett, J.R. Nery, M. Galli, A. Gallavotti and J.R. Ecker (2016) Cistrome and epicistrome features shape the regulatory DNA landscape. Cell 165: 1280–1292. 27203113 10.1016/j.cell.2016.04.038 PMC4907330

[B33] Pasquet, R.S. (2001) Notes on the genus *Vigna* (*Leguminosae-Papilionoideae*). Kew Bull 56: 223–227.

[B34] Patro, R., G. Duggal, M.I. Love, R.A. Irizarry and C. Kingsford (2017) Salmon provides fast and bias-aware quantification of transcript expression. Nat Methods 14: 417–419. 28263959 10.1038/nmeth.4197 PMC5600148

[B35] Paysan-Lafosse, T., M. Blum, S. Chuguransky, T. Grego, B.L. Pinto, G.A. Salazar, M.L. Bileschi, P. Bork, A. Bridge, L. Colwell et al. (2023) InterPro in 2022. Nucleic Acids Res 51: D418–D427. 36350672 10.1093/nar/gkac993 PMC9825450

[B36] Pertea, G. and M. Pertea (2020) GFF utilities: GffRead and GffCompare. F1000Res 9: 304. 10.12688/f1000research.23297.1 PMC7222033 32489650

[B37] Robinson, M.D., D.J. McCarthy and G.K. Smyth (2010) edgeR: a Bioconductor package for differential expression analysis of digital gene expression data. Bioinformatics 26: 139–140. 19910308 10.1093/bioinformatics/btp616 PMC2796818

[B38] Sakai, H., K. Naito, E. Ogiso-Tanaka, Y. Takahashi, K. Iseki, C. Muto, K. Satou, K. Teruya, A. Shiroma, M. Shimoji et al. (2015) The power of single molecule real-time sequencing technology in the de novo assembly of a eukaryotic genome. Sci Rep 5: 16780. 26616024 10.1038/srep16780 PMC4663752

[B39] Takahashi, Y., P. Somta, C. Muto, K. Iseki, K. Naito, M. Pandiyan, S. Natesan and N. Tomooka (2016) Novel genetic resources in the genus *Vigna* unveiled from gene bank accessions. PLoS One 11: e0147568. 26800459 10.1371/journal.pone.0147568 PMC4723357

[B40] Vanholme, R., I. Cesarino, K. Rataj, Y. Xiao, L. Sundin, G. Goeminne, H. Kim, J. Cross, K. Morreel, P. Araujo et al. (2013) Caffeoyl shikimate esterase (CSE) is an enzyme in the lignin biosynthetic pathway in Arabidopsis. Science 341: 1103–1106. 23950498 10.1126/science.1241602

[B41] Vaser, R., I. Sović, N. Nagarajan and M. Šikić (2017) Fast and accurate de novo genome assembly from long uncorrected reads. Genome Res 27: 737–746. 28100585 10.1101/gr.214270.116 PMC5411768

[B42] Voichek, Y., G. Hristova, A. Mollá-Morales, D. Weigel and M. Nordborg (2024) Widespread position-dependent transcriptional regulatory sequences in plants. Nat Genet 56: 2238–2246. 39266765 10.1038/s41588-024-01907-3 PMC11525189

[B43] Wada, Y., L.P.H. van Beek, C.M. van Kempen, J.W.T.M. Reckman, S. Vasak and M.F.P. Bierkens (2010) Global depletion of groundwater resources. Geophys Res Lett 37: L20402.

[B44] Wang, F., Y. Iki, K. Tanoi and K. Naito (2024) Phenotypic responses in the root of salt-tolerant accessions of *Vigna marina* and *Vigna luteola* under salt stress. Genet Resour Crop Evol 71: 2631–2640.

[B45] Wang, F., K. Tanoi, T. Yamauchi and K. Naito (2025) Multi-layered apoplastic barrier underlying the ability of Na^+^ exclusion in *Vigna marina*. Plant Cell Physiol 66: 554–565. 39777497 10.1093/pcp/pcaf005

[B46] Wehrens, R. and L.M.C. Buydens (2007) Self-and super-organizing maps in R: The kohonen package. J Stat Softw 21: v021i05.

[B47] Yan, D.H., T. Fenning, S. Tang, X. Xia and W. Yin (2012) Genome-wide transcriptional response of *Populus euphratica* to long-term drought stress. Plant Sci 195: 24–35. 22920996 10.1016/j.plantsci.2012.06.005

[B48] Yang, C., X. Li, S. Chen, C. Liu, L. Yang, K. Li, J. Liao, X. Zheng, H. Li, Y. Li et al. (2023) ABI5-FLZ13 module transcriptionally represses growth-related genes to delay seed germination in response to ABA. Plant Commun 4: 100636. 37301981 10.1016/j.xplc.2023.100636 PMC10721476

[B49] Yoshida, J., N. Tomooka, T.Y. Khaing, P.G.S. Shantha, H. Naito, Y. Matsuda and H. Ehara (2020) Unique responses of three highly salt-tolerant wild *Vigna* species against salt stress. Plant Prod Sci 23: 114–128.

[B50] Yu, Q., L. An and W. Li (2014) The CBL-CIPK network mediates different signaling pathways in plants. Plant Cell Rep 33: 203–214. 24097244 10.1007/s00299-013-1507-1

[B51] Zhang, J., W. Jia, J. Yang and A.M. Ismail (2006) Role of ABA in integrating plant responses to drought and salt stresses. Field Crops Res 97: 111–119.

