# Supplementary material for: Comparative genomics and transcriptomics on salt tolerance of Vigna luteola
Source: Breed Sci. 2026 Feb 4;76(2):144–54. doi: 10.1270/jsbbs.25042 (PMC13494335; doi:10.1270/jsbbs.25042)
Supplement: Supplementary file 1 [file 76_144_s1.pdf]

doi: 10.1270/jsbbs.25042

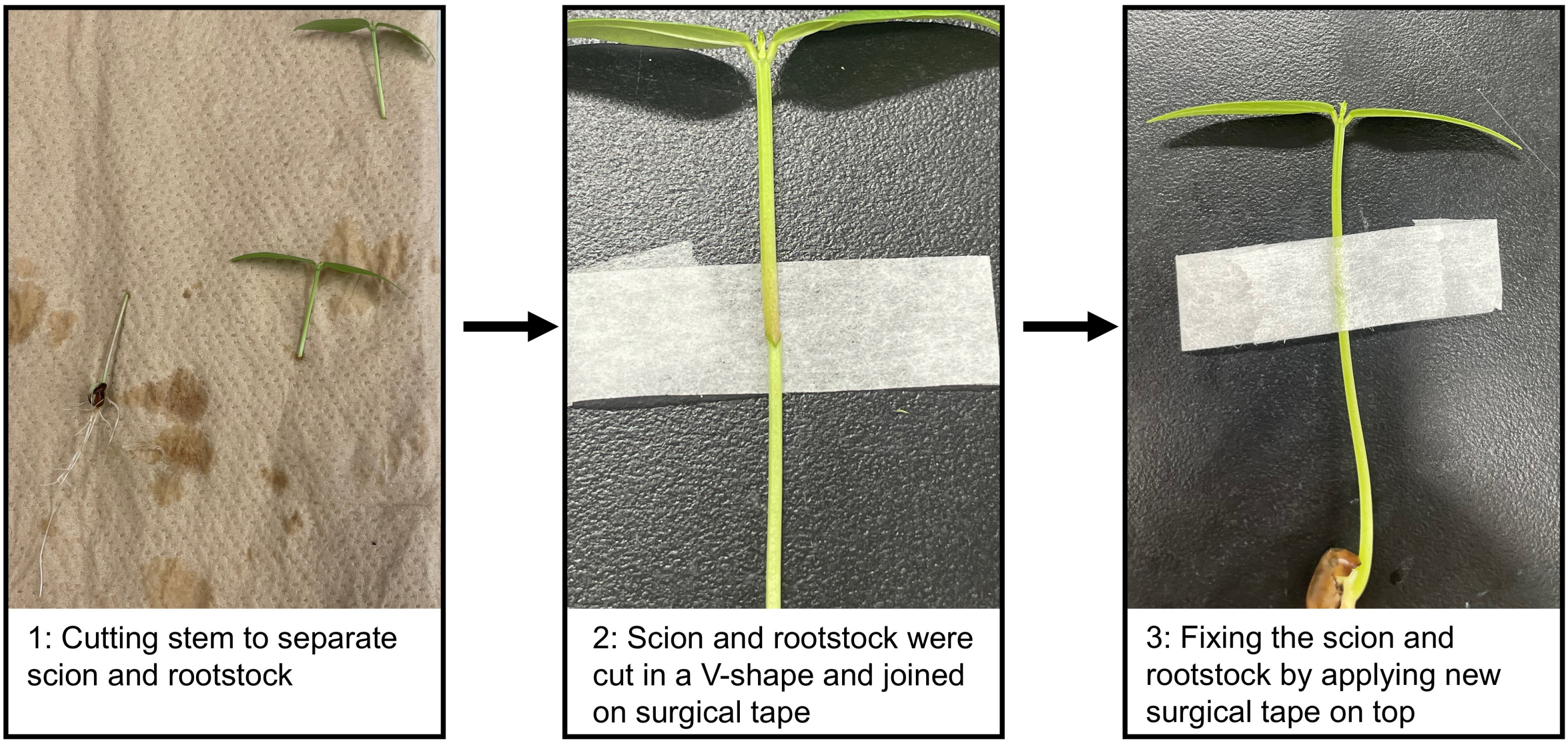

Supplemental Figure 1. Experimental procedure of grafting

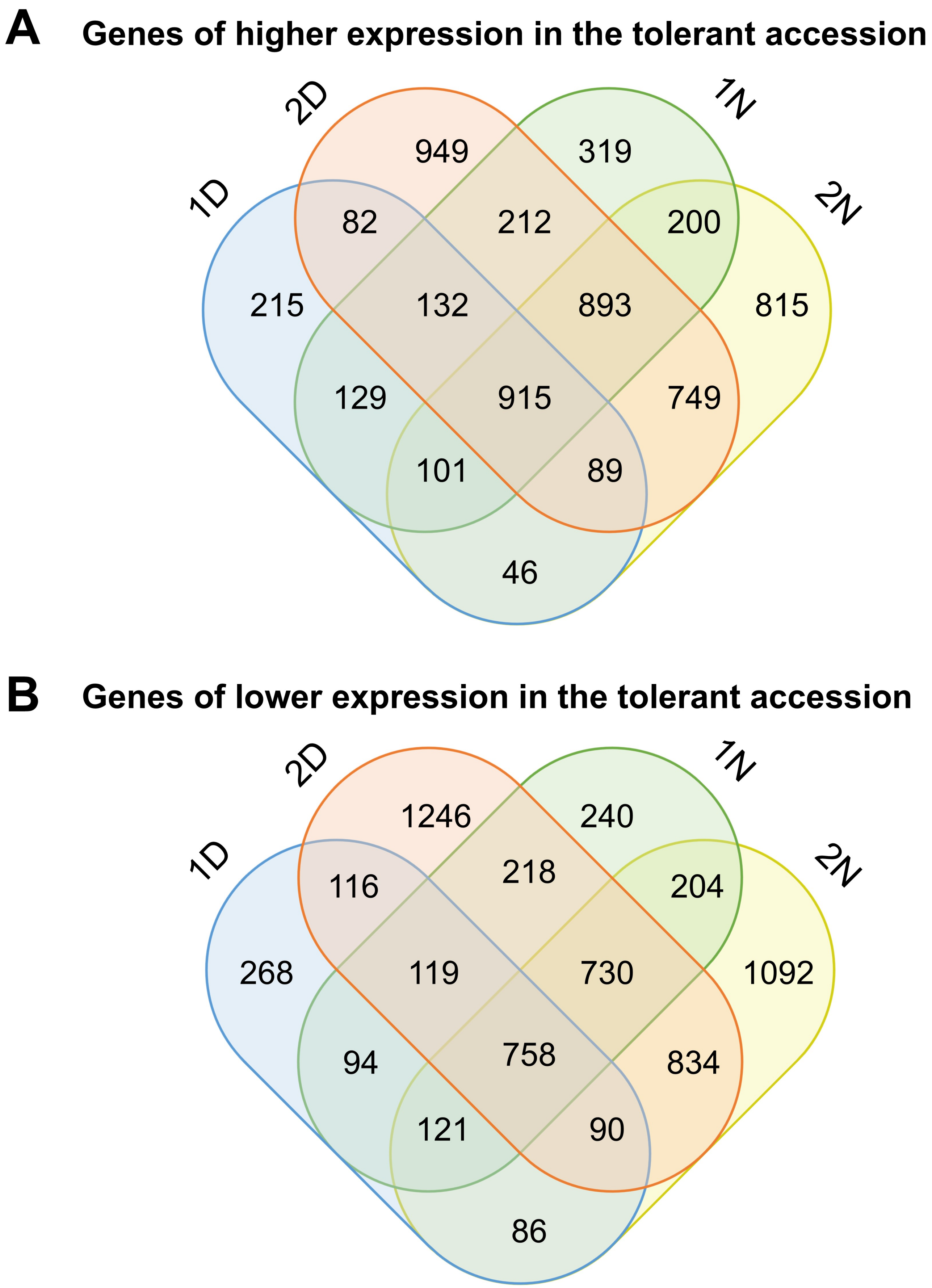

Supplemental Figure 2. The number of differentially expressed genes (DEGs) between the accessions under salt stress. (A) The number of genes with higher expression in the tolerant accession compared to the sensitive accession under salt stress. (B) The number of genes with lower expression in the tolerant accession compared to the sensitive accession under salt stress. DEGs were identified at each time point ($p < 0.05$ and FDR < 0.05). 1D, 1N, 2D, and 2N means 1st daytime, 1st nighttime, 2nd daytime, and 2nd nighttime, respectively. 6,917 genes detected at least 2 time points were used for further clustering.

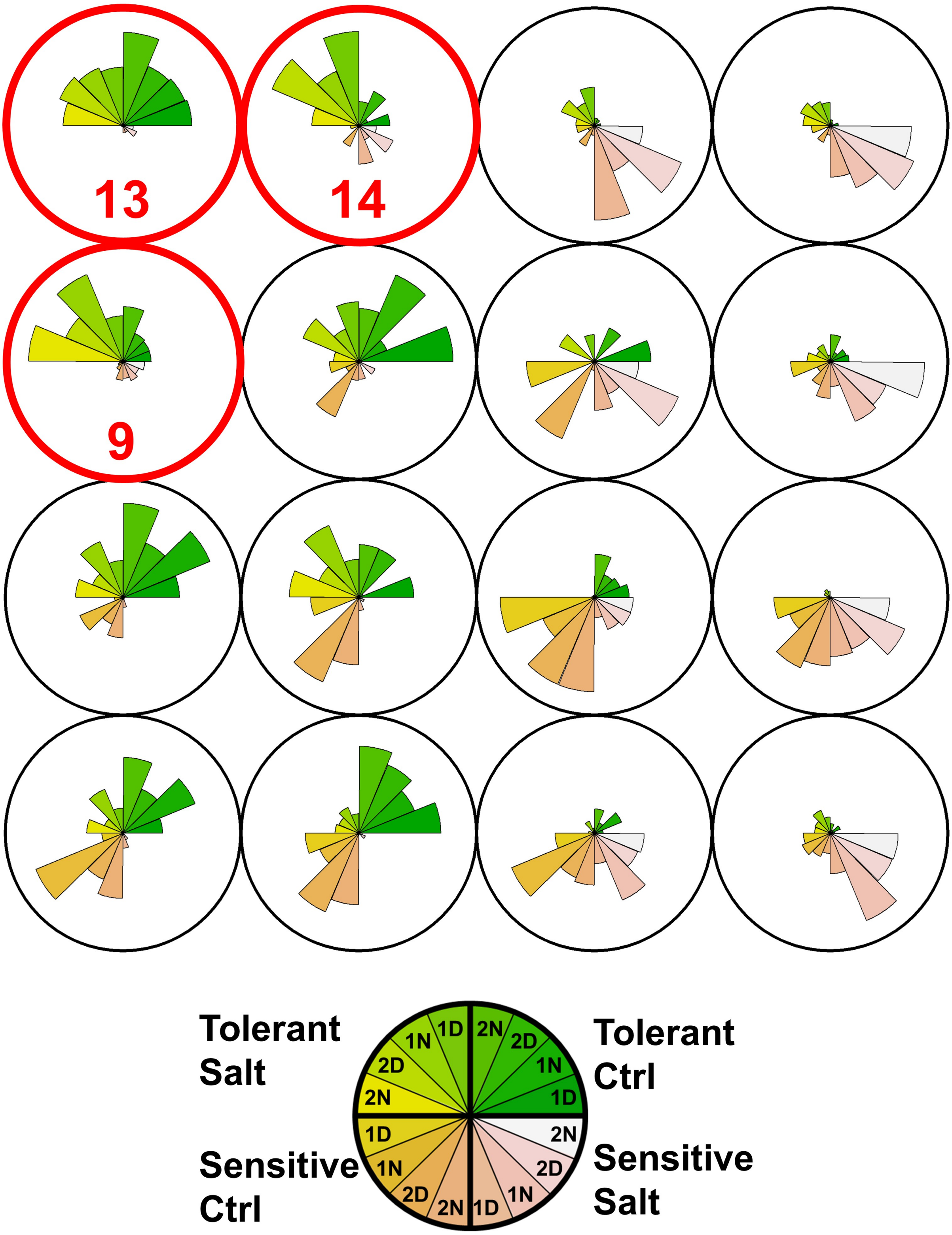

Supplemental Figure 3. The expression patterns of clusters generated with DEGs. Sixteen clusters were generated using 6,917 DEGs. The candidate clusters used in the subsequent analysis are indicated by red circles, and the red numbers represent the cluster IDs (e.g., "9" for cluster 9). In each cluster, the upper half is the tolerant accession, with the control condition on the right and the salt stress condition on the left. The lower half is the sensitive accession, with the control condition on the left and the salt stress condition on the right. The height of the bar is the relative expression of each time point. Tolerant and Sensitive indicate the tolerant accession and the sensitive accession, respectively. Ctrl and Salt indicate control and salt stress conditions, respectively. 1D, 1N, 2D, and 2N represent 1st daytime, 1st nighttime, 2nd daytime, and 2nd nighttime, respectively.

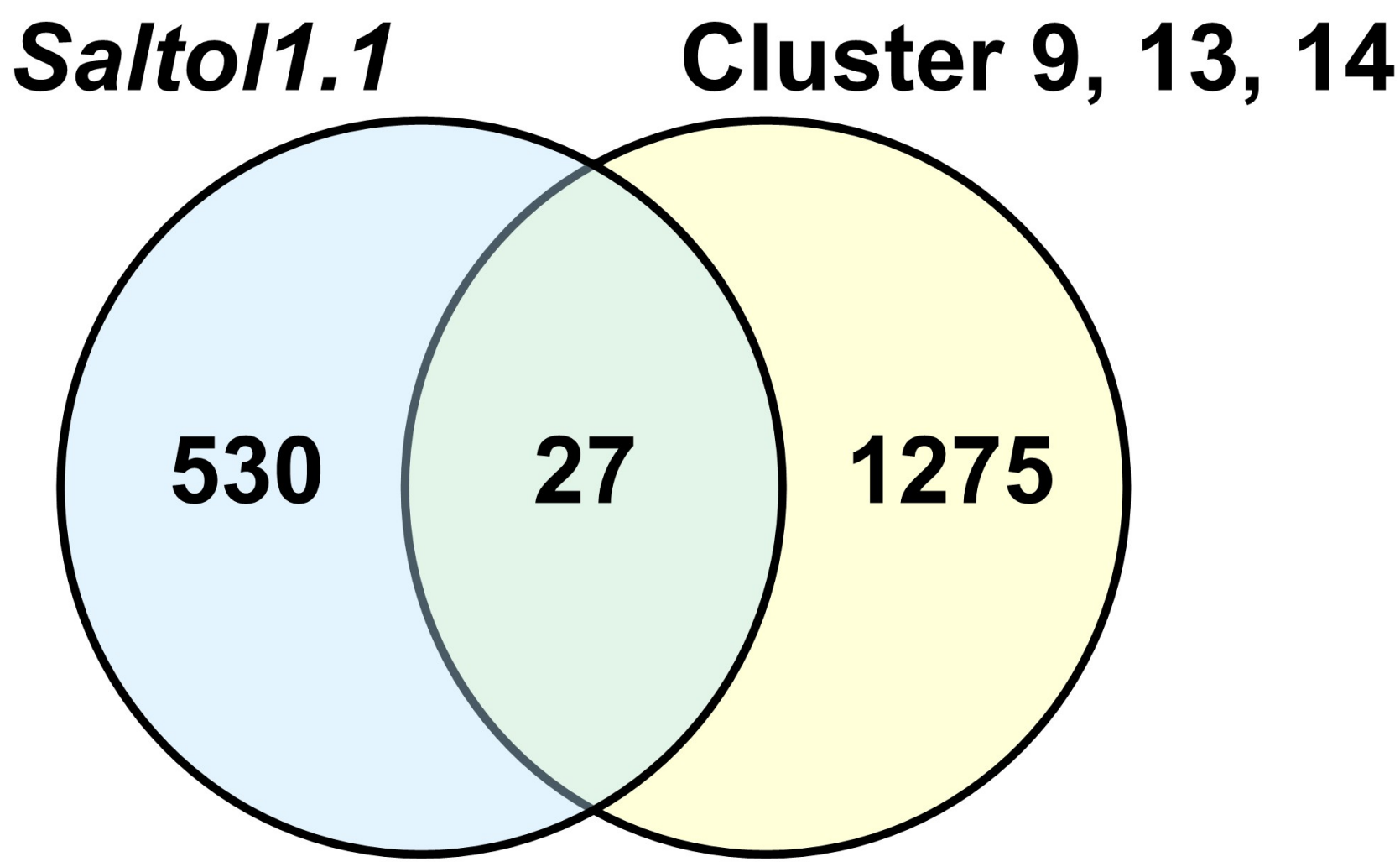

Supplemental Figure 4. Number of genes in the QTL Region and Candidate Clusters.

```
Viglu256296.01G0396300.1     1 MSEKEKEKEKEKEKGVGEGQTTLLHGKYELGRVLGHGTFAKVYHARNLKTGQHVAMKVVG
Viglu256418.01G0428000.1     1 ----MSEKEKEKEKGVGEGQTTLLHGKYELGRVLGHGTFAKVYHARNLKTGQHVAMKVVG

Viglu256296.01G0396300.1    61 KEKVIKVGMMEQVKREISVMKMVKHSNIVELHEVLASKSKIYIAMELVRGGELFNKVSKG
Viglu256418.01G0428000.1    57 KEKVIKVGMMEQVKREISVMKMVKHSNIVELHEVLASKSKIYIAMELVRGGELFNKVSKG

Viglu256296.01G0396300.1   121 RLKEDVARLYFQQLISAVDFCHSRGVYHRDLKPENLLLDEHGNLKVSDFGLTAFSDHLKE
Viglu256418.01G0428000.1   117 RLKEDVARLYFQQLISAVDFCHSRGVYHRDLKPENLLLDEHGNLKVSDFGLTAFSDHLKE

Viglu256296.01G0396300.1   181 DGLLHTTCGTPAYVSPEVIAKKGYDGAKADIWSCGVILYVLLAGFLPFQDDNLVAMYKKI
Viglu256418.01G0428000.1   177 DGLLHTTCGTPAYVSPEVIAKKGYDGAKADIWSCGVILYVLLAGFLPFQDDNLVAMYKKI

Viglu256296.01G0396300.1   241 YRGDFKCPPWFSAEARRLVTRLLDPNPNTRISISKVMESSWFKKPVPR--------RVEE
Viglu256418.01G0428000.1   237 YRGDFKCPPWFSAEARRLVTRLLDPNPNTRISISKVMESSWFKKPVPRRVEEVAKVDLEE

Viglu256296.01G0396300.1   293 VAKVDLEETCERPATMNAFHIISLSEGFDLSPLFEEKKREEMRFATAGTPSSVISRLEEV
Viglu256418.01G0428000.1   297 EAKVDLEETCERPATMNAFHIISLSEGFDLSPLFEEKKREEMRFATAGTPSSVISRLEEV

Viglu256296.01G0396300.1   353 AKAGKFDVKSSETKVRLQGQERGRKGKLAIAADIYAVTPSFMVVEVKKDNGDTLEYNQFC
Viglu256418.01G0428000.1   357 AKAGKFDVKSSETKVRLQGQERGRKGKLAIAADIYAVTPSFMVVEVKKDNGDTLEYNQFC

Viglu256296.01G0396300.1   413 SKQLRPALKDIFWNSPQNSTPAIVLE
Viglu256418.01G0428000.1   417 SKQLRPALKDIFWNSPQNSTPAIVLE
```

Supplemental Figure 5. Comparison of CIPK6 amino acid sequence between the tolerant accession and the sensitive accession. Viglu256296.01G0396300.1 and Viglu256418.01G0428000.1 are CIPK6 homologs of the tolerant and the sensitive accessions, respectively. Letters on a black background indicate amino acids that are identical between the two accessions. Letters on a grey background indicate strongly conserved amino acid groups. The orange and blue underlines indicate the position of the kinase and NAF domains, respectively.

```
Viglu256296.01G0424800.1     1 MLGKRPRPMIGKLSELLVSRGRAVTFLDP----ATATAGSPKTPSGTAAMQSPRGFKNYH
Viglu256418.01G0456600.1     1 MLGKRPRPMIGKLSELLVSRCRAVTLLDPATATATATAGSPKTPSGTAAMQSPRGFKDYH

Viglu256296.01G0424800.1    57 LGGVGLGIVVALDKPSNEVLPKHAVCTSHLKIQDEFDVEEYTYVTRHVPNKTLTKVYYDG
Viglu256418.01G0456600.1    61 LGGVGLGIVVALDKPSNEVLPKHAVCTSHLKIQDEFDVEEYTYVTRHVPNKTLTKVYYDG

Viglu256296.01G0424800.1   117 GEGEIQTHDYSNNNINTNKNKNNLGVFRRTAPQPLFESESSYPTSNFLSSCNLCGKKLHG
Viglu256418.01G0456600.1   121 GEGEIQTHDYSNNNINTNKNKNNLGVFRRTAPQPLFESESSYPTSNFLSSCNLCGKKLHG

Viglu256296.01G0424800.1   177 KDIYMYRGEKAFCSPECRSSQIMMDERKERCRSEASRSVEMSSSPYTREHIFSTGILAL
Viglu256418.01G0456600.1   181 KDIYMYRGEKAFCSPECRSSQIMMDERKERCRSEASRSVEMSSSPYTREHIFSTGILAL
```

Supplemental Figure 6. Comparison of FLZ13 amino acid sequence between the tolerant and the sensitive accessions. Viglu256296.01G0424800.1 and Viglu256418.01G0456600.1 are FLZ13 homologs of the Tolerant and Sensitive accession, respectively. Letters on a black background indicate amino acids that are identical between the two accessions. The orange underline indicates the position of the zf-FLZ domain.

A

```
Viglu256296.01G0445600.1     1 MSSSLSQAKSLRLLLPKSISLHRRGYAVASDISARVGLGNNIGRRSGIVGGAEEKPVTRD
Viglu256418.01G0478400.1     1 MSSSLSQAKSLRLLLPKSISLHRRGYAVASDISARVGLGNNIGRRSGIVGGAEEKPVTRD

Viglu256296.01G0445600.1    61 GAKAYSDWAPDPVTGDYRPINHTPEIDPVELRRMLLNHKFRSPQ
Viglu256418.01G0478400.1    61 GAKAYSDWAPDPVTGDYRPINHTPEIDPVELRRMLLNHKFRSPQ
```

B

```
Viglu256296.01G0413000.1     1 MAPEPPSSEAPPNFWGHMPEEEYYTSQGVRNTKSHFQTPNGKIFTQSFLPLHQPIKATVF
Viglu256418.01G0444900.1     1 MAPEPPSSEAPPNFWGHMPEEEYYTSQGVRNTKSHFQTPNGKIFTQSFLPLHQPIKATVF

Viglu256296.01G0413000.1    61 MTHGYGSDTGWLFQKICINFANWGYAVFAADLLGHGRSEGLRCYLGDMETVAATSLAFFL
Viglu256418.01G0444900.1    61 MTHGYGSDTGWLFQKICINFANWGYAVFAADLLGHGRSEGLRCYLGDMETVAATSLAFFL

Viglu256296.01G0413000.1   121 DVRRSEPYKTLPAFLFGESMGGLATLLMYFQSDPGTWTGLIFSAPLFVIPEDMKPSKVHL
Viglu256418.01G0444900.1   121 DVRRSEPYKTLPAFLFGESMGGLATLLMYFQSDPGTWTGLIFSAPLFVIPEDMKPSKVHL

Viglu256296.01G0413000.1   181 FVYGLLFGLADTWAAMPDNKMVGKAIRDPEKLKVIASNPRRYTGPPRVGTMRELLRVTQY
Viglu256418.01G0444900.1   181 FVYGLLFGLADTWAAMPDNKMVGKAIRDPEKLKVIASNPRRYTGPPRVGTMRELLRVTQY

Viglu256296.01G0413000.1   241 VQDNFSRVTTPFFTAHGTADGVTCPSSSKLLYEKASSEDKTLKLYDGMYHSLIQGEPDES
Viglu256418.01G0444900.1   241 VQDNFSRVTTPFFTAHGTADGVTCPSSSKLLYEKASSEDKTLKLYDGMYHSLIQGEPDES

Viglu256296.01G0413000.1   301 ANLVLGDMREWIDERVRRYGS
Viglu256418.01G0444900.1   301 ANLVLGDMREWIDERVRRYGS
```

Supplemental Figure 7. Comparison of DI21 and CSE amino acid sequences between the Tolerant and Sensitive accessions. (A) Viglu256296.01G0445600.1 and Viglu256418.01G0478400.1 are DI21 homologs of the tolerant and the sensitive accessions, respectively. (B) Viglu256296.01G0413000.1 and Viglu256418.01G0444900.1 are CSE homologs of the tolerant and the sensitive accessions, respectively. Letters on a black background indicate amino acids that are identical between the two accessions.

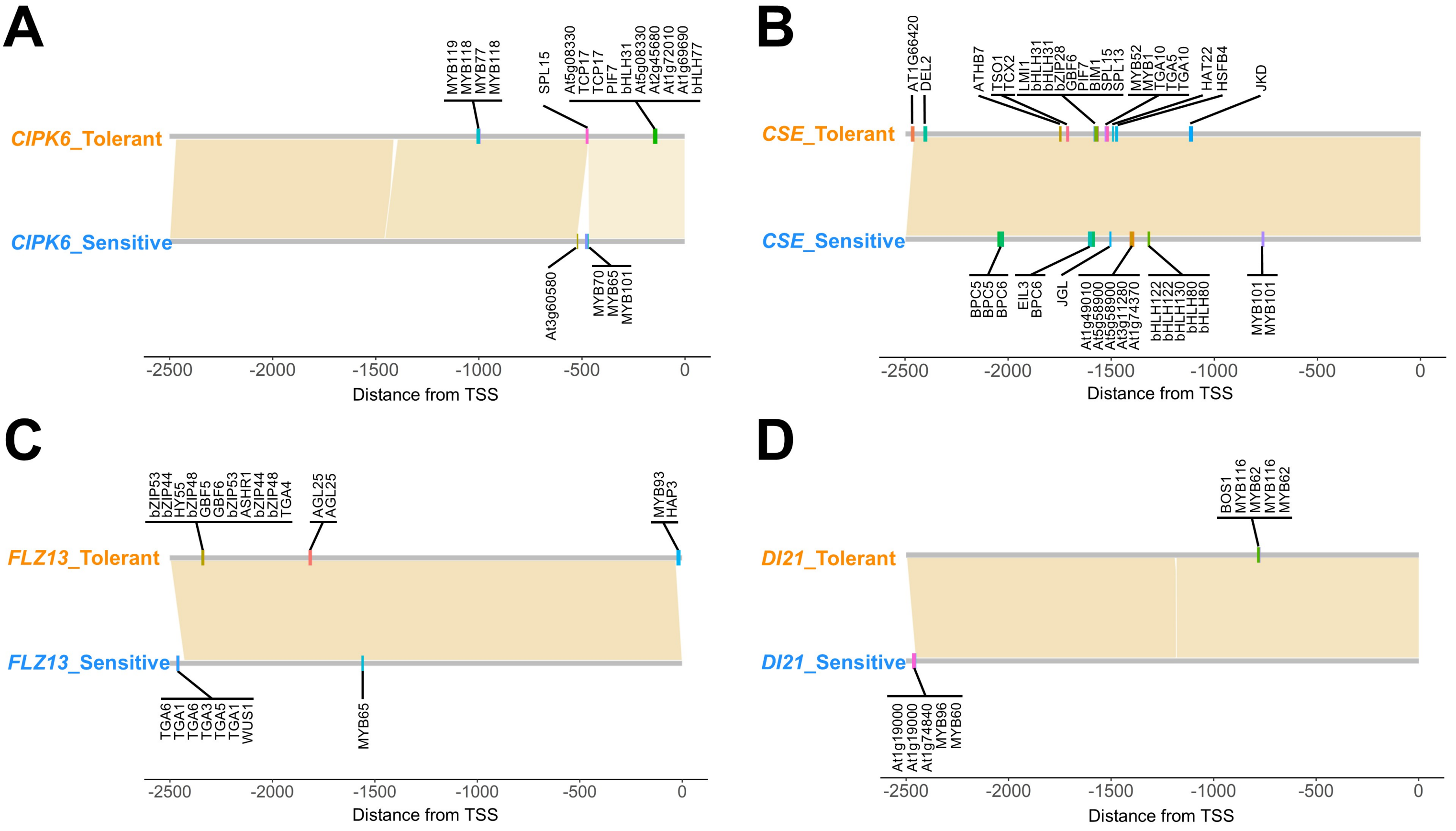

Supplemental Figure 8. Positions of motifs unique to the tolerant or the sensitive accessions in the promoter regions of candidate genes. (A), (B), (C), and (D) shows the results of *CIPK6*, *CSE*, *FLZ13*, and *DI21,* respectively. The same motif may appear more than once within a promoter region. In this figure, we show all appearances, even if the same motif type occurs multiple times. In the main text, however, the numbers refer to distinct motif types, with each type counted only once per promoter. Homologous regions between the accessions, detected using BLASTN, are highlighted. The highlighted regions are adapted from a Kablammo-generated figure with slight modifications.

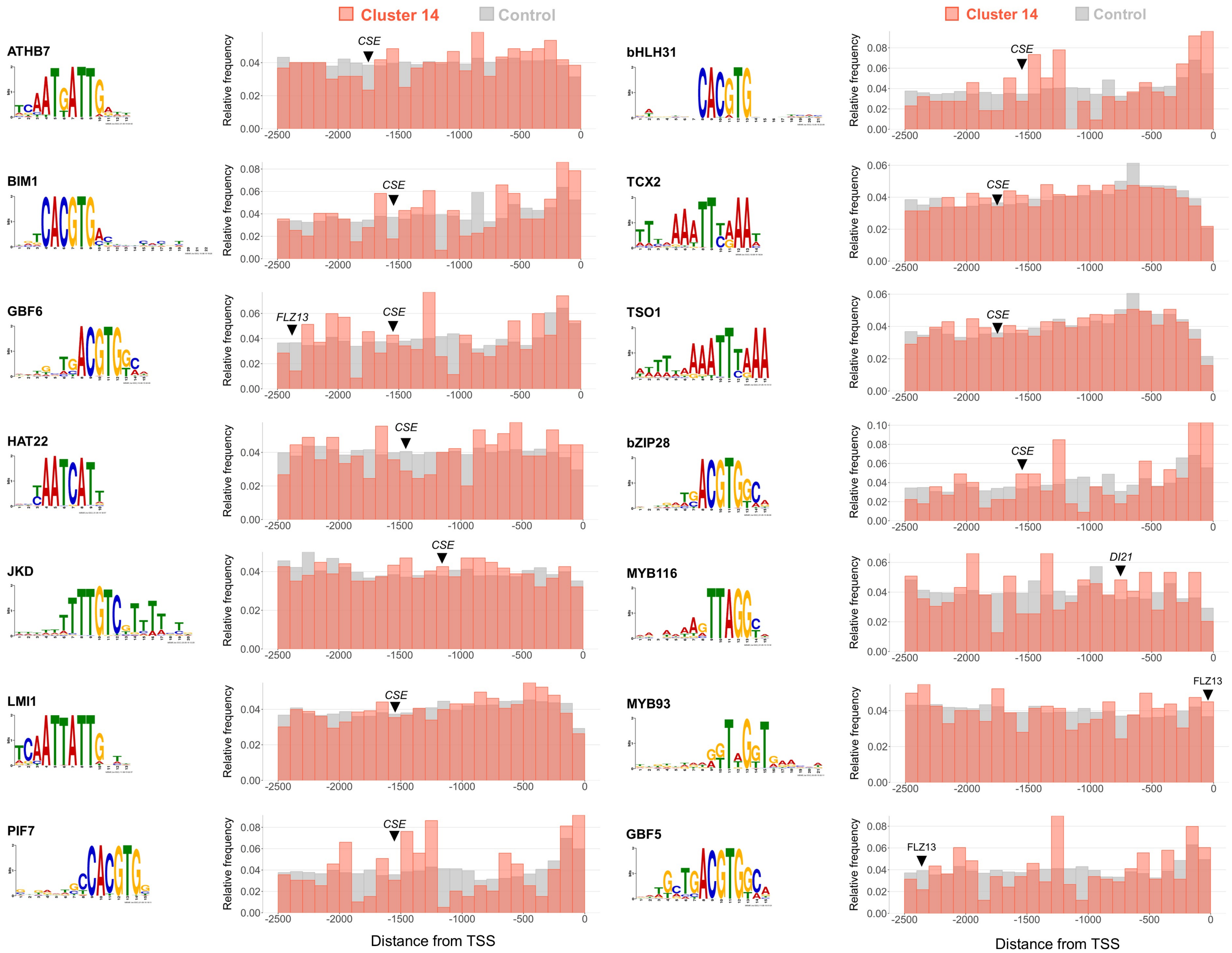

Supplemental Figure 9. Transcription factor binding motifs unique to the tolerant accession in the *CSE*, *DI21*, and *FLZ13* promoters, enriched in cluster 14 promoters compared to control. Pictograms of the motifs and relative frequencies of these motifs across the promoters of cluster 14 and control groups are shown. Black arrows indicate the positions of the motifs on the *CSE*, *DI21*, and *FLZ13* promoters.

A

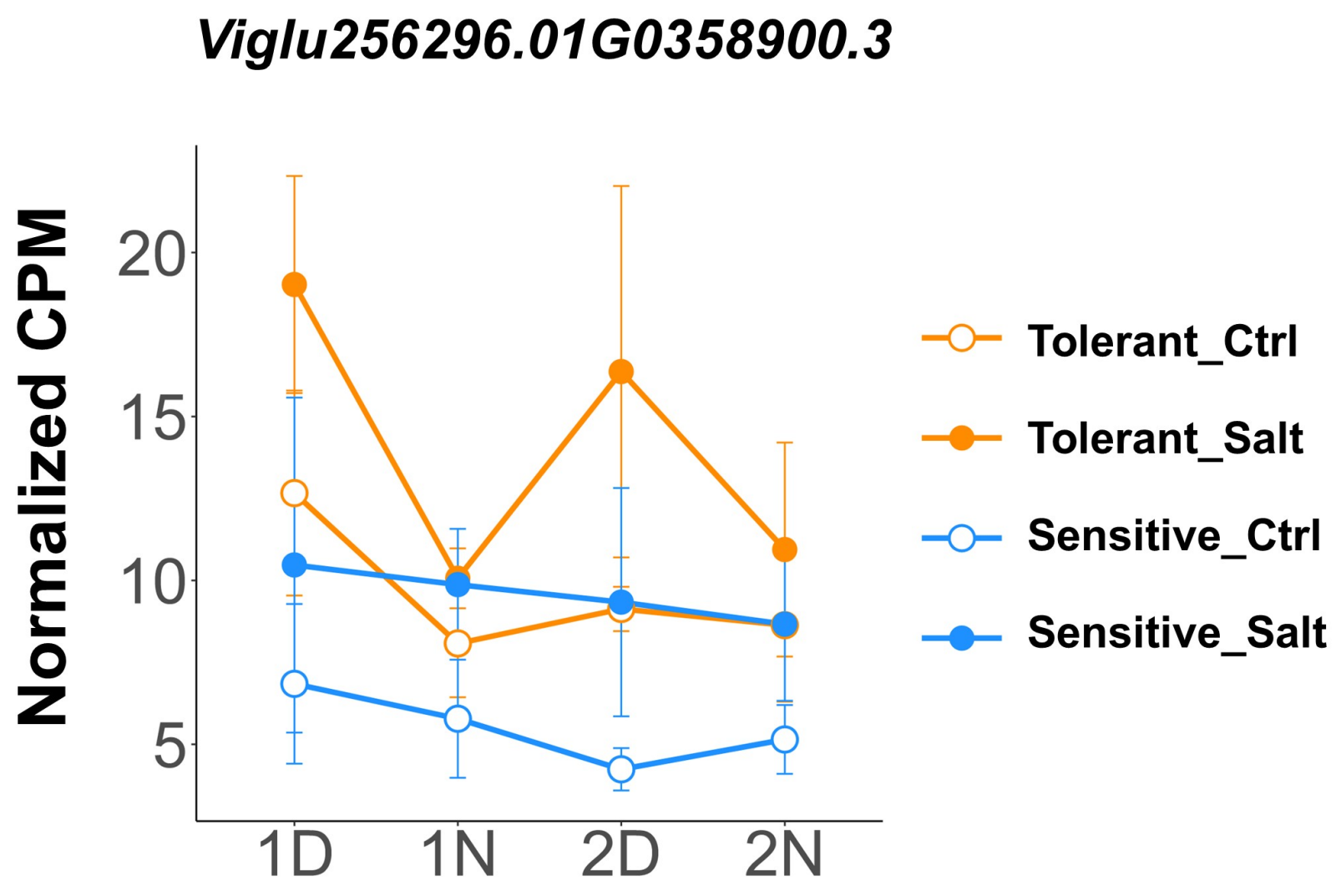

B

```
Viglu256296.01G0358900.3    1 -----------------MELPLINDATFSSANPSLSEIWPSQFPSSNKRPSSLLLHNHSP
AT1G59640.2                 1 MDPSGMMNEGGPFNLAEIWQFPLNGVSTAGDSSRRSFVGPNQFGDADLTTAANGDPARMS

Viglu256296.01G0358900.3   44 NSNKHVKLSPPS--------------------------ETHQNAALQAPPPDATSVAAAH
AT1G59640.2                61 HALSQAVIEGISGAWKRREDESKSAKIVSTIGASEGENKRQKIDEVCDGKAEAESLGTET

Viglu256296.01G0358900.3   78 KSPLQTPKPKQDYIHVRARRGQATDSHSLAERARREKISERMKILQDLVPGCSKVIGKAL
AT1G59640.2               121 EQKKQQMEPTKDYIHVRARRGQATDSHSLAERARREKISERMKILQDLVPGCNKVIGKAL

Viglu256296.01G0358900.3  138 VLDEIINYIQSLQRQVEFLSMKLEAINSRLNMSPSIESFPSKEVATQPFDLSGIIFG---
AT1G59640.2               181 VLDEIINYIQSLQRQVEFLSMKLEAVNSRMNPGIEVFPPKEVMILMIINSIFSIFFTKQY

Viglu256296.01G0358900.3  195 ---------------------------------------------SQPVREYAQGSQAGW
AT1G59640.2               241 MFLSRYSRGRSLDVYAVRSFKHCNKRSDLCFCSCSPKTELKTTIFSQNMTCFCRYSRVGV

Viglu256296.01G0358900.3  210 LHMQIAGGFEKVT------------------------------
AT1G59640.2               301 AISSSKHCNEPVTLCFYSYCLRKIYHFLLWNLKYKIQKSVLFS
```

Supplemental Figure 10. Expression pattern and amino acid sequence of Viglu256296.01G0358900.3 (A) Expression levels of *Viglu256296.01G0358900.3*, a homolog bHLH31. (B) Comparison of amino acid sequence between Viglu256296.01G0358900.3 and AT1G59640.2 (bHLH31). Letters on a black background indicate amino acids that are identical between the two accessions. Letters on a grey background indicate strongly conserved amino acid groups. The orange lines indicate the position of the bHLH_AtBPE_like domains for each sequence.
